# FOXP3 Polymorphism and Upregulation of the CXCL12‐CXCR4‐SNAIL Axis with High Infiltration of M2TAM by STAT3/NFKB Pathways Influence the Survival of Cervical Cancer Patients

**DOI:** 10.1002/adbi.202500354

**Published:** 2025-11-20

**Authors:** George A. Lira, Fábio M. de Azevedo, Ingrid G. S. Lins, Janaína C. O. Crispim, Giovanna A. Lira, Rômulo S. Cavalcante, Ricardo Cobucci, Carolina O. Mendes‐Aguiar, Rafaela Torres Dantas Da Silva, Vinícius E. da Silva, Ryan C. Q. Aquino, Raimundo F. Araújo Júnior

**Affiliations:** ^1^ Cancer and Inflammation Research Laboratory Department of Morphology Federal University of Rio Grande do Norte Natal RN 59072‐970 Brazil; ^2^ Post‐Graduation in Health Science Federal University of Rio Grande do Norte Natal RN 59072‐970 Brazil; ^3^ League Against Cancer from Rio Grande do Norte Advanced Oncology Center Natal RN 59075‐740 Brazil; ^4^ Pathology Department Federal University of Rio Grande do Norte Natal RN 59012‐570 Brazil; ^5^ Post‐Graduate Program in Technological Development and Innovation in Medicines Federal University of Rio Grande do Norte Natal RN 59072‐970 Brazil; ^6^ Trairi Faculty of Health Sciences Federal University of Rio Grande do Norte Santa Cruz RN 59200‐000 Brazil; ^7^ Graduate Program of Biotechnology Potiguar University (UnP) Natal RN 59056‐000 Brazil; ^8^ Laboratory of Immunogenetics of Complex Diseases Institute of Tropical Medicine of Rio Grande do Norte, UFRN Natal RN 59072‐970 Brazil

**Keywords:** cytokines, immune response, immunotherapy, survival, tumor progression

## Abstract

This study explores the interaction between immune and cancer cells in the tumor microenvironment (TME) of cervical carcinoma (CC), with emphasis on tumor‐associated macrophages (M2‐TAMs) and the STAT3‐NF‐κB signaling pathway. It investigates how Treg cell polymorphisms and TAM infiltration through these pathways influence overall survival (OS) in CC patients. This prospective study follows 100 CC patients from 2018 to 2023 using qRT‐PCR and immunohistochemistry on tumor samples, and flow cytometry on blood samples to evaluate immunosuppressive cytokines and Treg cell polymorphisms. High stromal CD163+204+ TAM density, mediated by STAT3/NF‐κB, correlates with biomarkers such as Ki‐67, VEGFα, and FOXP3 (*p* < 0.001). XPO5 expression is associated with increased STAT3, SNAIL, and HPV 16/18 levels. FOXP3 T allele deletion and HLA‐G polymorphism in the blood of patients correlate with higher STAT3 tumor expression and elevated IL‐4 and IL‐17 blood cytokines. The CXCL12‐CXCR4 axis shows a strong association with STAT3, SNAIL in TME and blood cytokines, including IL‐6 and IL‐12. Elevated CXCL12, CXCR4, and SNAIL expression in TME significantly increases mortality risk. These findings underscore the role of M2TAM infiltration and immune modulation in tumor progression and clinical outcomes in CC.

## Graphical abstract

This study highlights how cervical carcinoma progression is shaped by immune–tumor interactions within the TME. M2‐TAM infiltration, regulated through STAT3/NF‐κB and linked to CXCL12–CXCR4, promotes immune suppression, angiogenesis, and poor survival. Associations with Treg polymorphisms, HLA‐G variants, and cytokine shifts emphasize the interplay between genetic and microenvironmental factors, underscoring novel prognostic and therapeutic targets.

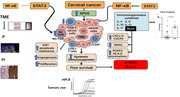

## 1 Introduction

Cervical cancer is the fifth most common disease among women worldwide and a significant public health issue, particularly in underdeveloped countries with high incidence and mortality rates.^[^ ^1^, ^2^, ^3^ ^]^ The immune system plays a dual role in cancer progression, acting as both a defender and a facilitator of tumor initiation. Many tumors evade immune surveillance by downregulating MHC class I, impairing antigen processing, resisting apoptosis, and attracting immunosuppressive cells.^[^ ^4^ ^]^ Within the tumor microenvironment (TME), tumor‐associated 2 macrophages (M2‐TAMs) actively promote tumor growth, vascularization, and stromal modulation through STAT3/NF‐κB pathway interactions.^[^ ^5^, ^6^, ^7^ ^]^

Cervical cancer is strongly linked to persistent infection by carcinogenic Human Papillomavirus (HPV) types 16 and 18.^[^ ^8^ ^]^ While the immune system can typically clear the virus, ≈10% of cases persist, altering crucial genes by mutations which lead to premalignant lesions such as low‐ and high‐grade squamous intraepithelial lesions (LSIL and HSIL).^[^ ^8^, ^9^ ^]^ Furthermore, oncogenes play a crucial role in immune response regulation in TME.^[^ ^4^, ^10^ ^]^ One of them is Exportin 5 (XPO5), a transporter of pre‐miRNAs, which facilitates the expression of CXCL12, CXCR4, SNAIL, and FOXP3, promoting immunosuppression, tumor progression, and metastasis, while also influencing tumor recurrence following standard therapies.^[^ ^11^, ^12^, ^13^, ^14^ ^]^ Other oncogenes release regulatory and immunosuppressive proteins that drive epithelial‐mesenchymal transition (EMT), proliferation, and angiogenesis, ultimately leading to poor survival in solid tumors.^[^ ^15^, ^16^ ^]^ These proteins include vascular endothelial growth factor (VEGF), tumor growth factor β (TGFβ), vimentin (VIM), E‐cadherin (E‐cad), IL‐10, CD25, metalloproteinases (MMPs), macrophage inhibitory factor (MIF), and IL‐17.^[^ ^4^, ^17^, ^18^ ^]^

Additionally, human leukocyte antigen (HLA)‐G, a non‐classical MHC Ib antigen with a 14‐bp insertion/deletion polymorphism in the 3′ untranslated region (3′‐UTR), is associated with immune escape, metastasis, and poor prognosis in several cancers.^[^ ^19^, ^20^ ^]^ The role of HLA‐G in cervical cancer pathogenesis remains unclear, though previous studies suggest that it induces the formation of FOXP3+ tumor‐infiltrating Treg cell, which inhibits immune response by down‐regulating the anti‐tumor T cell proliferation.^[^ ^21^ ^]^ In this context, FOXP3 polymorphisms have also been associated with the development and poor prognosis of cervical, gastric, and colorectal cancers.^[^ ^22^ ^]^ Understanding the complex interactions between stromal and cancer cells in the TME, along with identifying biomarkers that predict metastasis, is essential for developing personalized immunotherapy strategies.^[^ ^23^ ^]^

For the first time, this study demonstrates that increased expression of CXCL12‐CXCR4 and SNAIL, along with high infiltration of M2‐TAMs in a +HPV/XPO5 TME and an activated STAT3/NF‐κB signaling pathway, is significantly associated with higher mortality risk in cervical cancer patients with FOXP3/HLA‐G polymorphisms.

## 2 Experimental Section

### 2.1 Tissue Samples and Data Collection

**Figure 1:**
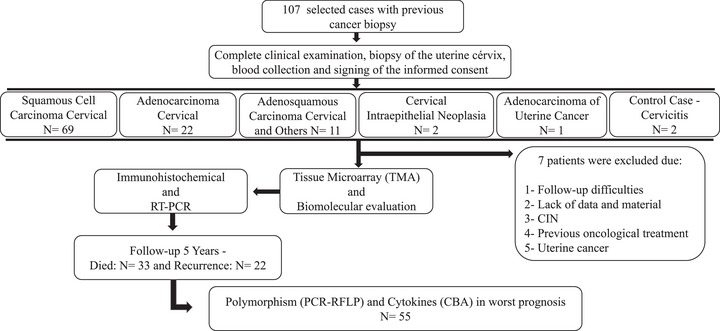
Flowchart of the study design for cervical cancer patients (2018–2023). A total of 107 patients were selected and followed for five years, with seven excluded for not meeting predetermined criteria. Analyses included Tissue Microarray, Immunohistochemistry, Real‐Time Quantitative Reverse Transcription Polymerase Chain Reaction (RT‐PCR), Polymerase Chain Reaction‐Restriction Fragment Length Polymorphism (PCR‐RFLP), Flow Cytometry, and Cervical intraepithelial neoplasia (CIN).

This prospective study analyzed 100 women diagnosed with cervical cancer between 2018 and 2023, registered at the Advanced Oncology Center of the League Against Cancer of Rio Grande do Norte (LRNCC), Natal, Brazil. After excluding seven patients (**Figure** **1**), the inclusion criteria required: 1) no prior oncological treatment, 2) TNM staging system classification, 3) physical ability to provide tumor and blood samples, 4) signed Informed Consent Form (ICF), 5) no surgical contraindications affecting prognosis, and 6) sufficient surgical material for tissue microarray (TMA).^[^ ^24^ ^]^ Approved by the LRNCC Research Ethics Committee (IRB) (CAE 2 761 333, Natal, Brazil) on May 9, 2018, the study followed institutional guidelines, with patient treatment managed by the responsible physician.

### 2.2 Tissue Microarray (TMA) Block Construction and Staining by Hematoxylin & Eosin (H&E)

Tissue Microarray (TMA) blocks were constructed as described by Araujo Jr. et al., 2015.^[^ ^25^ ^]^ Briefly, all hematoxylin & eosin (H&E) slides were re‐examined by a second experienced pathologist. Two morphologically representative fields of invasive cervical cancer were selected and marked. Two 1 mm‐diameter samples from each neoplasm, obtained from biopsies and/or surgical specimens, were extracted from donor blocks and transferred to a recipient block using the Manual Tissue Microarrayer I (Beecher Instruments, Silver Spring, USA). Three‐micrometer sections of the microarray block were cut using the Paraffin Tape‐Transfer System (Instrumedics, Saint Louis, USA). One section was stained with H&E to confirm the presence of the tumor by light microscopy. To enhance the reliability of the results, immunohistochemical reactions were performed on two separate slides. The histological sections were placed on 8% silanized glass slides for immunohistochemical analysis.

### 2.3 Immunofluorescence (IF) and Immunohistochemistry (IHC)

To better understand the interaction between the STAT3 signaling pathway and M2‐TAMs (CD204) in the TME of CC, which represents one of the central focuses of the study, double immunofluorescence staining was performed on TMA slides using anti‐STAT3 and anti‐CD204 antibodies (Supporting Information).^[^ ^25^, ^26^, ^27^, ^28^ ^]^ Furthermore, Immunohistochemical analysis of TMA slides was conducted as described by Araújo Jr. et al. (2016) in a cohort of 95 patients.^[^ ^29^ ^]^ Using a microtome, 4‐µm‐thick tumor sections were mounted onto gelatin‐coated slides. The paraffin‐embedded tissues were incubated at 4 °C for 60 min or overnight with primary antibodies targeting CD25, CD163, IL‐10, TGFβ, VEGFα, IL‐17, NF‐κB, PD‐L1, E‐Cadherin, Bcl‐2, MMP9, Ki‐67, Vimentin, CD204, SNAIL, MIF, STAT3, FOXP3, and P16 (Table S1, Supporting Information). After rinsing in PBS, sections were treated with a streptavidin/HRP‐conjugated secondary antibody (Biocare Medical, Concord, CA, USA), and immunoreactivity was visualized using a colorimetric detection kit (TrekAvidin‐HRP Label + Kit, Biocare Medical, Pacheco, USA), following the manufacturer's protocol. Positive and negative controls were routinely included.

For P16, immunohistochemistry was performed using a mouse monoclonal antibody on the Ventana Benchmark GX automated immunostainer (Tucson, AZ, USA) following standard protocols.^[^ ^30^ ^]^ High‐resolution images were acquired at 200× and 400× magnifications using an Aperio AT2 slide scanner (Aperio, Vista, CA, USA) and processed with ImageScope software.

### 2.4 Quantitative Evaluation of Immunostaining

Staining intensity was considered positive when cells exhibited brown staining in the nucleus, cytoplasm, and/or membrane (Table S2, Supporting Information). Marker expression was quantified using numerical results from software‐based image analysis algorithms.^[^ ^31^, ^32^ ^]^ The analysis yielded a median value between color intensity and the number of positively stained cells (Supporting Information).^[^ ^33^, ^34^, ^35^ ^]^ A hotspot was defined as the area with the highest density of Ki‐67‐positive tumor cells. Ki‐67 expression was categorized into low‐ and high‐proliferation groups, with cutoff values of up to 15% and greater than 15%, respectively (Supporting Information).^[^ ^36^ ^]^

### 2.5 Cytokine Analysis by Flow Cytometry

Cytokine analysis was performed on 55 patients who died or experienced recurrence over a five‐year follow‐up (2018–2023). Levels of Interleukin 2 (IL‐2), Interleukin 4 (IL‐4), Interleukin 6 (IL‐6), Interleukin 10 (IL‐10), Tumor Necrosis Factor (TNF), Interferon‐γ (IFN‐γ), and Interleukin 17A (IL‐17A) were measured using flow cytometry (Cytoflex, Beckman Coulter) with the BD Cytometric Bead Array (CBA) Human Th1/Th2/Th17 Cytokine Kit (California, USA). Samples were analyzed in duplicate and compared with controls from 10 cancer‐free individuals.^[^ ^37^ ^]^

The standard mixture was reconstituted per the manufacturer's instructions, yielding a 5000 pg mL^−1^ stock concentration for each cytokine. Serial dilutions (0–5000 pg mL^−1^) were prepared in an assay buffer. Samples were incubated with antibody‐coated microspheres for 3 h at room temperature, then washed and resuspended in a wash buffer. Flow cytometry quantified labeled proteins based on phycoerythrin fluorescence. Cytokine concentrations were automatically calculated using FCAP Array software with a standard curve from recombinant cytokines.

Results were categorized into two groups: Group 1—values below the minimum detection threshold, and Group 2—values within the standard curve (Table S3, Supporting Information).^[^ ^38^ ^]^

### 2.6 Real‐Time Quantitative Reverse Transcription Polymerase Chain Reaction (RT‐PCR)

Total RNA was extracted from 100 cervical cancer patients using TRIzol reagent (Invitrogen, USA) and purified with the SV Total RNA Isolation System (Promega, USA) following the manufacturer's protocol. cDNA synthesis was performed with the High‐Capacity RNA‐to‐cDNA Kit (Applied Biosystems, USA), and real‐time amplification was conducted using PowerUp SYBR Green Master Mix (Applied Biosystems, USA). Nine samples were excluded due to excessive cDNA contamination, resulting in a final analysis of 91 cervical cancer samples and 2 non‐tumor cervical tissue samples (negative controls).

Gene expression analysis was performed on non‐transformed (negative control, *n* = 2) and malignantly transformed (cervical cancer, *n* = 91) cervical tissue samples. Samples were stored in Trizol at −80 °C until analysis, then thawed and homogenized. RNA extraction was carried out using chloroform and ethanol, followed by purification with the SV Total RNA Isolation System (Promega, USA). The purified RNA was reverse transcribed into cDNA using the High‐Capacity RNA‐to‐cDNA Kit (Applied Biosystems, USA).

Real‐time quantitative PCR (qPCR) was performed using PowerUp SYBR Green Master Mix (Applied Biosystems) with forward and reverse primers (Thermo Fisher Scientific, listed in Table S4, Supporting Information). All experiments were conducted in triplicate under standard qPCR conditions: 50 °C for 2 min, 95 °C for 10 min, followed by 40 cycles of 94 °C for 30 s, a variable annealing temperature for 30 s, and 72 °C for 1 min. The average Ct values were used to determine the relative expression of target genes, normalized to the housekeeping gene β‐actin using the 2‐ΔΔCt method. Gene expression changes were classified as decreased (Rq ≤ 1.0), increased (Rq > 1.0), or unchanged (Rq = 1.0)

### 2.7 Genotyping for Polymerase Chain Reaction‐Restriction (PCR) and Fragment Length Polymorphism (PCR‐RFLP)

Whole blood was collected from 55 patients who died or experienced recurrence, and plasma was separated by centrifugation.^[^ ^39^ ^]^ Genomic DNA (gDNA) was extracted for FOXP3 and HLA‐G polymorphism analysis using the salting‐out method (Salazar et al., with modifications).^[^ ^40^ ^]^ DNA quality and integrity were verified on a 1% agarose gel and quantified using a Nanodrop spectrophotometer (Thermo Scientific‐GE). The β‐globin gene was amplified in parallel with each sample as an internal control.

HLA‐G 14‐bp Ins/Del genotyping (rs1704) in the 3′‐UTR of exon 8 was performed using PCR. A 25 µL reaction mixture contained 200 ng of gDNA, 0.2 mm dNTPs, 0.2 mm primers, 0.5 U Taq DNA polymerase, 1.5 mm MgCl2, and 1× PCR buffer. The thermal cycling program included an initial denaturation at 94 °C for 5 min, followed by 30 cycles at 95 °C for 45 s, 56 °C for 45 s, and 72 °C for 30 s, with a final extension at 72 °C for 10 min. Amplification products were analyzed on a 3.5% agarose gel, with a 345‐bp fragment indicating the deletion allele and a 359‐bp fragment indicating the insertion allele.^[^ ^19^ ^]^

Genotyping of FOXP3 rs3761549 SNPs was performed using the PCR‐RFLP method (Gomes et al.).^[^ ^37^ ^]^ PCR amplification used the following primers:

- Forward: 5′‐GCCTGGCACTCTCAGAGCTT‐3′
- Reverse: 5′‐TGCCCACGGTCCAGAAATAC‐3′

The PCR thermal program included an initial denaturation at 94 °C for 5 min, followed by 35 cycles of 94 °C for 1 min, 60 °C for 1 min, and 72 °C for 1 min, with a final extension at 72 °C for 10 min. PCR products were visualized on a 2% agarose gel. Next, 5 µL of PCR products were digested in an 11 µL restriction enzyme reaction mixture containing 0.5 µL (10 U µL^−1^) of BseNI, 1 µL of 10× PCR buffer, and 9.5 µL of dH2O, incubated at 65 °C for 12 h. Digested products were separated on a 2% agarose gel by electrophoresis and visualized under UV light. The banding pattern identified the genotypes:

- TT: 525, 383, and 35 bp
- CC: 525, 213, 170, and 35 bp
- CT: 525, 383, 213, 170, and 35 bp.^[^ ^42^ ^]^

HLA‐G alleles were classified as insertion (I) or deletion (D), forming II, DD, and DI genotypes (20). FOXP3 genotypes were categorized based on T and C alleles, with TT, CC, and CT combinations.^[^ ^43^ ^]^

### 2.8 Follow‐Up

Overall survival (OS) was defined as the time from the date of diagnosis or the start of treatment until death or the last follow‐up. The analysis included a total of 100 patients with cancer, of whom 67 were alive and 33 had died.^[^ ^44^, ^45^ ^]^ (Table S5, Supporting Information)

### 2.9 Statistical Analysis

A descriptive analysis of the variables was conducted using frequency distribution tables for nominal variables, while quantitative variables were summarized using statistical measures such as mean, standard deviation, minimum, maximum, and median.

To evaluate potential dependencies between molecular markers, clinical staging, polymorphisms, and cytokines, multiple Chi‐Square tests of independence were performed. The null hypothesis (H0) assumed no relationship between the variables, while the alternative hypothesis (H1) suggested a dependency. These tests were applied to 2 × 2 contingency tables. Additionally, for polymorphism analysis, odds ratios were calculated to quantify and estimate the likelihood of events associated with allele expression.

Survival analysis was performed using the Kaplan–Meier method to estimate survival time until death and compare survival between independent groups. The log‐rank test was used to assess differences between survival curves and determine the influence of specific factors. A significance level of 0.05 was established, with p‐values below this threshold considered statistically significant.

All statistical analyses were carried out using IBM SPSS Statistics 24.0 (Chicago, IL). Additionally, a non‐parametric Spearman correlation analysis was conducted to assess relationships between gene markers (RT‐PCR) and blood cytokine levels, given their non‐Gaussian distribution (*p* < 0.05, two‐tailed, 95% confidence interval).

### 2.10 Ethics Statement

This study was approved by the LRNCC Research Ethics Committee (IRB) (CAE 2761333, Natal, Brazil) on May 9, 2018.

## 3 Results

### 3.1 Epidemiologic and Clinicopathologic Features of the 100 Cervical Cancer Patients

The characteristics of 100 patients are summarized in Tables S5 and S6 (Supporting Information). The average age of the patients was 47.17 years, with 63 (63%) individuals falling within this range (Table S5, Supporting Information).

Histological analysis was performed to assess tumor differentiation and aggressiveness. Of the total patients, 47 had poorly differentiated tumors. In terms of clinical staging, 34 patients were classified as stage I, 21 as stage II, and 44 as stage III or IV, while staging information was unavailable for one patient. Additionally, 33 patients succumbed to the disease, and 22 experienced tumor recurrence.

Regarding initial treatment, 39 patients underwent surgery. Among them, 7 (17.94%) presented with angiolymphatic invasion, 9 (23.07%) had neural invasion, and 21 (79.48%) exhibited full stromal invasion. Lymph node metastasis was detected in 12 (30.76%) patients. The average number of resected retroperitoneal lymph nodes was 2.5. The mean number of pelvic lymph nodes removed from the right and left iliac fossae was 3.4 and 3.2, respectively, with one positive lymph node found on both sides (Table S6, Supporting Information).

### 3.2 Overall Survival to Age, Epidemiologic Data, Laboratory Analysis, and ClinicoPathological Features

As shown in Table S7 (Supporting Information), a univariate Log‐Rank logistic analysis was applied to the TNM staging system in the investigated population. The relative risks (chi‐square) for patients from stage I to stage III + IV were 22.13, with median survival times of 40.0 months (35.0–46.0) for stage I, 40 months (35.0–43.0) for stage II, and 40 months (31.0–43.0) for stages III + IV, all with significant *p*‐values (*p* < 0.001).

Furthermore, a statistically significant correlation was observed between survival and several laboratories and clinicopathological features, including hemoglobin levels (g dL^−1^, ≥10 vs <10; *p* < 0.05), surgical treatment (yes vs no; *p* < 0.05), degree of pathological differentiation (*p* < 0.05), and TNM pathological staging (stage I vs II and stage II vs III–IV; *p* < 0.001) (Table S7, Supporting Information). However, survival did not show a statistically significant correlation with age, certain epidemiological factors, or some laboratory and clinicopathological parameters (Tables S7, Supporting Information).

### 3.3 STAT3, NF‐κB, CD204 and CD163 Protein Expression, and Its correlation of Association

**Figure 2:**
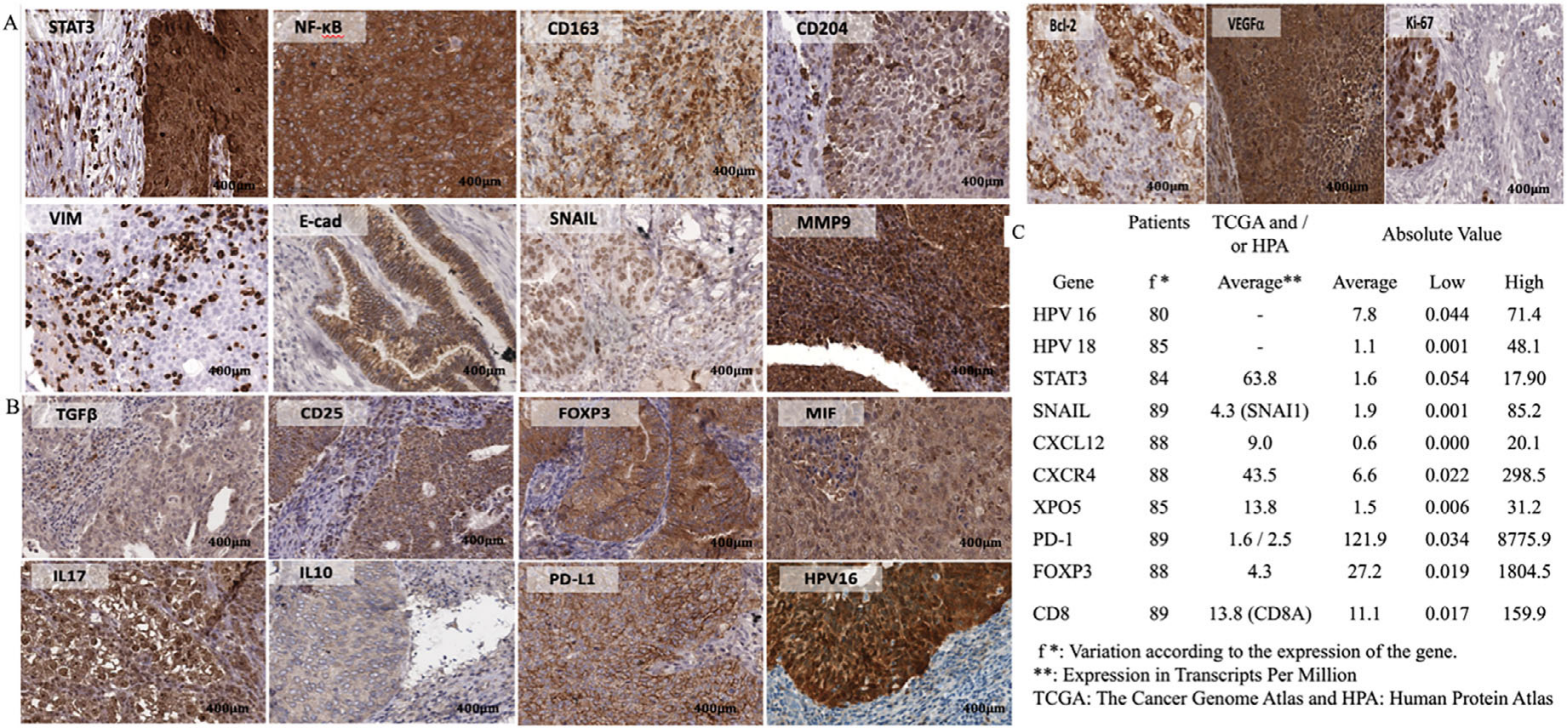
Protein and gene profiles of tumor progression markers in 100 patients with cervical cancer. A,B) Tissue Microarray (TMA) and Immunohistochemical analysis (×40, scale 0.05 µm). A) Strong cytoplasmic/nuclear staining for STAT3, NF‐κB, CD163, CD204, KI‐67, and MMP9. VEGFα showed strong cytoplasmic staining. VIM and E‐cadherin exhibited cytoplasmic/membrane staining, while ki‐67 and SNAIL showed strong nuclear staining. B) Strong cytoplasmic staining was observed for TGFβ, CD25, FOXP3, MIF, IL‐17, IL‐10, and PD‐L1. HPV16 demonstrated strong cytoplasmic/nuclear staining in TMA. C) Gene expression profile of STAT3, SNAIL, PD‐1, HPV16, HPV18, FOXP3, XPO5, CXCR4, CXCL12, and CD8 by rt‐PCR. ^*^f (variation according to the expression of the gene and ^**^ Public genes).

First, to evaluate the interaction between STAT3 and CD204 expression in the TME of cervical cancer (CC) patients, we performed double immunofluorescence staining with anti‐STAT3 and anti‐CD204 antibodies on TMA sections from 100 cervical cancer patients. Quantitative assessment revealed co‐expression of STAT3 and CD204 in 56 patients, indicating a moderate/strong correlation between these markers (Figure S1, Supporting Information). To examine protein expression levels, we evaluated cervical cancer tissues using immunohistochemistry (IHC). STAT3 and CD204 proteins were primarily observed in the cytoplasm and nucleus, whereas NF‐κB and CD163 proteins were mainly localized in the cytoplasm (**Figure** **2**A–C).

First, we assessed whether the cellular expression of CD204+ or CD163+ M2‐TAM correlated with the STAT3/NF‐κB signaling pathways in the tumor microenvironment (TME) of 95 patients with cervical cancer (**Table** **1** and Figure 2). A statistically significant correlation was observed between CD163 and CD204 expression and NF‐κB (*p* < 0.05), with strong immunoexpression detected in 65 and 80 patients, respectively (Table 1).

**Table 1:** Protein expressions of STAT3, NF‐κB, CD163, and CD204 in cervical cancer and the correlation of association between them.

			STAT3			NF‐κB			CD163		
Marker	Immuno‐ Expression		1		2			1	2		
	Immuno‐ Expression 1		Weak n [%]	Strong n [%]	*p*	Weak n [%]	Strong n [%]	*p*	1	2	
			Weak	7 (30.4)	16 (69.6)	<0.001	–	–	Weak n [%]	Strong n [%]	*p*
NF‐κB	2	Strong	1 (1.4)	71 (98.6)		–	–	–	–	–	–
	1	Weak	8 (26.7)	22 (73.3)	<0.001	14 (46.7)	16 (53.3)		–	–	
CD163	2	Strong	0 (0.0)	65 (100.0)		9 (13.8)	56 (86.2)	<0.05	–	–	
	1	Weak	5 (41.7)	7 (58.3)	<0.001	6 (50.0)	6 (50.0)		–	–	
CD204	2	Strong	3 (3.6)	80 (96.4)		17 (20.5)	66 (79.5)	<0.05	9 (75.0)	3 (25.0)	<0.05

*p* = Significant *p* < 0.05 (Chi‐square Test), and 5 patients did not undergo immunohistochemistry.

Additionally, STAT3/NF‐κB transcription factors showed a significant correlation with each other (*p* < 0.001 and *p* < 0.05) (Table 1 and Figure 2A–C).

### 3.4 Evaluation of Epithelial‐Mesenchymal Transition (EMT) by Vimentin, E‐Cadherin, SNAIL, and MMP9 Protein Expression, and Its Correlation of Association with STAT3, NF‐κB, CD163, and CD204 Protein Expression

In order to study the tumor progression, including EMT, sustained by upregulation of CD 163 and CD 204 (M2‐TAM) and STAT3, NF‐κB in TME of 100 patients with CC¸, we analyzed the protein expression of these markers by IH. First, we observed that VIM and E‐cad proteins were expressed mainly in the cytoplasm / membrane, SNAIL was observed mainly in the nuclear, and MMP9 protein was mainly in the cytoplasm / nuclear. The strong staining of VIM, E‐cad, SNAIL and MMP9 was seen in 89.5%, 80.0%, 91.6% and 96.8% of the cases, respectively (**Tables** **2** and **3**, and Figure 2A–C).

**Table 2:** Protein expression of EMT, apoptosis, angiogenesis, and proliferation, and its correlation of association with STAT3/NF‐κB signaling pathways.

Marker	Immuno‐ Expression	STAT3			NF‐κB		
		Weak n [%]	Strong n [%]	p	Weak n [%]	Strong n [%]	p
VIM	Weak	4 (40.0)	6 (60.0)	<0.001	4 (40.0)	6 (60.0)	0.21
	Strong	4 (4.7)	81 (95.3)		19 (22.4)	66 (77.6)	
E‐cad	Weak	6 (31.6)	13 (68.4)	<0.001	10 (52.6)	9 (47.4)	<0.05
	Strong	2 (2.6)	74 (97.4)		13 (17.1)	63 (82.9)	
SNAIL	Weak	3 (37.5)	5 (62.5)	<0.05	3 (37.5)	5 (62.5)	0.35
	Strong	5 (5.7)	82 (94.3)		20 (23.0)	67 (77.0)	
MMP9	Weak	2 (66.7)	1 (33.3)	<0.05	2 (66.7)	1 (33.3)	0.08
	Strong	6 (6.5)	86 (93.5)		21 (22.8)	71 (77.2)	
HPV16	Weak	2 (8.0)	23 (92.0)	0.90	5 (20.0)	20 (80.0)	0.52
	Strong	6 (8.8)	62 (91.2)		18 (26.5)	50 (73.5)	
Bcl‐2	Weak	4 (57.1)	3 (42.9)	<0.05	6 (85.7)	1 (14.3)	<0.001
	Strong	4 (4.5)	84 (95.5)		17 (19.3)	71 (80.7)	
VEGFα	Weak	4 (50.0)	4 (50.0)	<0.001	4 (50.0)	4 (50.0)	0.07
	Strong	4 (4.6)	83 (95.4)		19 (21.8)	68 (78.2)	
Ki‐67	Weak	3 (60.0)	2 (40.0)	<0.001	3 (60.0)	2 (40.0)	0.05
	Strong	5 (5.6)	85 (94.4)		20 (22.2)	70 (77.8)	

*p* = Significant *p* < 0.05 (Chi‐square Test), and 5 patients did not undergo immunohistochemistry.

VIM, E‐cad, MMP9, and SNAIL had high expression for strong marking and were correlated with STAT3 and CD204 (Tables 2 and 3). The expression of VIM (*p* < 0.001), E‐cad (*p* < 0.001), SNAIL (*p* < 0.05), and MMP9 (*p* < 0.05) was statistically significant with the expression of STAT3 (Table 2). While E‐cad had significance with NF‐κB (*p* < 0.05) e CD163 (*p* > 0.05). On the other side, VIM, E‐cad, SNAIL and MMP9 had significance with CD204 (**Table** **4**).

**Table 3:** Protein expression of EMT, apoptosis, angiogenesis, and proliferation, and its correlation of association with M2‐TAM (CD163 and CD204).

Marker	Immuno‐ Expression	CD163			CD204		
		Weak n [%]	Strong n [%]	p	Weak n [%]	Strong n [%]	p
VIM	Weak	5 (50.0)	5 (50.0)	0.18	6 (60.0)	4 (40.0)	<0.05
	Strong	25 (29.4)	60 (70.6)		6 (7.1)	79 (92.9)	
E‐cad	Weak	12 (63.2)	7 (36.8)	<0.05	7 (36.8)	12 (63.2)	<0.001
	Strong	18 (23.7)	58 (76.3)		5 (6.6)	71 (93.4)	
SNAIL	Weak	4 (50.0)	4 (50.0)	0.24	3 (37.5)	5 (62.5)	<0.05
	Strong	26 (29.9)	61 (70.1)		9 (10.3)	78 (89.7)	
MMP9	Weak	2 (66.7)	1 (33.3)	0.18	2 (66.7)	1 (33.3)	<0.05
	Strong	28 (30.4)	64 (69.6)		10 (10.9)	82 (89.1)	
HPV16	Weak	7 (28.0)	18 (72.0)	0.68	4 (16.0)	21 (84.0)	0.45
	Strong	22 (32.4)	46 (67.6)		7 (10.3)	61 (89.7)	
Bcl‐2	Weak	4 (57.1)	3 (42.9)	0.13	6 (85.7)	1 (14.3)	<0.001
	Strong	26 (29.5)	62 (70.5)		17 (19.3)	71 (80.7)	
VEGFα	Weak	6 (75.0)	2 (25.0)	<0.05	4 (50.0)	4 (50.0)	0.07
	Strong	24 (27.6)	63 (72.4)		19 (21.8)	68 (78.2)	
Ki‐67	Weak	4 (80.0)	1 (20.0)	<0.05	3 (60.0)	2 (40.0)	<0.05
	Strong	26 (28.9)	64 (71.1)		20 (22.2)	70 (77.8)	

*p*‐values < 0.05 were considered statistically significant (Chi‐square test). Five patients did not undergo immunohistochemistry.

**Table 4:** Protein expression of immunosuppression and its correlation of association with STAT3/NF‐κB signaling pathways.

Marker	Immuno‐ Expression	STAT3			NF‐κB		
	Immuno‐ Expression	Weak n [%]	Strong n [%]	p	Weak n [%]	Strong n [%]	p
TGFβ	Weak	7 (11.1)	56 (88.9)	0.18	17 (27.0)	46 (73.0)	0.37
	Strong	1 (3.1)	31 (96.9)		6 (18.8)	26 (81.3)	
CD25	Weak	7 (17.1)	34 (82.9)	<0.05	14 (34.1)	27 (65.9)	<0.05
	Strong	1 (1.9)	53 (98.1)		9 (16.7)	45 (83.3)	
FOXP3	Weak	4 (26.7)	11 (73.3)	<0.001	5 (33.3)	10 (66.7)	0.39
	Strong	4 (5.0)	76 (95.0)		18 (22.5)	62 (77.5)	
MIF	Weak	4 (66.7)	1 (33,3)	<0.001	6 (100.0)	0 (00.0)	<0.001
	Strong	4 (4.5)	85 (95.5)		17 (19.1)	72 (80.9)	
IL‐17	Weak	2 (50.0)	2 (50.0)	<0.05	3 (75.0)	1 (25.0)	<0.05
	Strong	6 (6.6)	85 (93.4)		20 (22.0)	71 (80.7)	
IL‐10	Weak	6 (9.1)	60 (90.9)	<0.001	18 (27.3)	48 (72.7)	0.29
	Strong	2 (6.9)	27 (93.1)		5 (17.2)	24 (82.8)	
PD‐L1	Weak	7 (9.9)	64 (90.1)	0.38	21 (29.6)	50 (70.4)	<0.05
	Strong	1 (4.2)	23 (95.8)		2 (29.6)	22 (91.7)	

*p*‐values < 0.05 were considered statistically significant (Chi‐square test). Five patients did not undergo immunohistochemistry.

### 3.5 Evaluation of Immunosuppression by TGFβ, CD25, FOXP3, MIF, IL‐17, IL‐10, PD‐L1 and HPV16 Protein Expression and Its Correlation of Association with STAT3, NF‐κB, CD204 and CD163 Protein Expression

The high infiltration of M2‐TAM (CD 163 and CD 204) and the upregulation of STAT3/NF‐κB pathways promote an immunosuppressive profile in the TME. In this sense, we studied the expression of several immunosuppression markers in the TME of 100 patients with CC. The expression of TGFβ, CD25, FOXP3, MIF, IL‐17, IL‐10, PD‐L1, and HPV16 was analyzed for correlation with STAT3, NF‐κB, CD163, and CD204 (Tables 4 and **5**, and Figure 2A–C). TGFβ, CD25, FOXP3, MIF, IL‐17, IL‐10, and PD‐L1 were primarily cytoplasmic, while HPV16 was detected in both cytoplasm and nucleus. Strong staining was observed in 33.7–95.8% of cervical cancer cases. FOXP3 and MIF expression correlated significantly with CD163 (*p* < 0.05), while CD204 was strongly associated with FOXP3 and MIF (*p* < 0.001). Higher expression of CD25 (*p* < 0.05), FOXP3 (*p* < 0.001), MIF (*p* < 0.001), IL‐17 (*p* < 0.05), and IL‐10 (*p* < 0.001) correlated with increased STAT3 expression. NF‐κB expression was significantly associated with CD25 (*p* < 0.05), MIF (*p* < 0.001), IL‐17 (*p* < 0.05), and PD‐L1 (*p* < 0.05).

**Table 5:** Protein expression of immunosuppression and its correlation of association with M2‐TAM (CD163 and CD204).

Marker	Immuno‐ Expression	CD163			CD204		
		Weak n [%]	Strong n [%]	p	Weak n [%]	Strong n [%]	p
TGFβ	Weak	22 (34.9)	41 (65.1)	0.32	10 (15.9)	53 (84.1)	0.18
	Strong	8 (25.0)	24 (75.0)		2 (6.3)	30 (93.8)	
CD25	Weak	17 (41.5)	24 (58.5)	0.07	8 (19.5)	33 (80.5)	0.07
	Strong	13 (24.1)	41 (75.9)		4 (7.4)	50 (92.6)	
FOXP3	Weak	13 (86.7)	2 (13.3)	<0.001	7 (46.7)	8 (53.3)	<0.001
	Strong	17 (21.3)	63 (78.8)		5 (6.3)	75 (93.8)	
MIF	Weak	4 (66.7)	2 (33.3)	0.05	4 (66.7)	2 (33.3)	<0.001
	Strong	26 (29.2)	63 (78.8)		8 (9.0)	81 (91.0)	
IL‐17	Weak	2 (50.0)	2 (50.0)	0.41	2 (50.0)	2 (50.0)	<0.05
	Strong	28 (30.4)	63 (69.2)		10 (11.0)	81 (89.0)	
IL‐10	Weak	23 (34.8)	43 (65.2)	0.30	10 (15.2)	56 (84.8)	0.26
	Strong	7 (24.1)	22 (75.9)		2 (6.9)	27 (93.1)	
PD‐L1	Weak	23 (32.4)	48 (67.6)	0.76	11 (15.5)	60 (84.5)	0.14
	Strong	7 (29.2)	17 (70.8)		1 (4.2)	23 (95.8)	

*p* = Significant *p*<0.05 (Chi‐square Test), and 5 patients did not undergo immunohistochemistry.

### 3.6 Evaluation of Apoptosis, Angiogenesis, and Proliferation by Bcl‐2, VEGFα, and Ki‐67 Protein expression, and Its Correlation of Association with STAT3, NF‐κB, CD204, and CD163 Protein Expression

The tumor progression, including resistance of apoptosis, angiogenesis, and proliferation, is strongly influenced by higher infiltration of M2‐TAM (CD163 and 204) and upregulation of STAT3, NF‐κB pathways. Based on this evidence, we analyzed the expression of Bcl‐2, VEGF, and Ki‐67 in the TME of 100 patients with CC.

The marking of protein expression was, mainly, cytoplasmic/nuclear to Bcl‐2, cytoplasmic to VEGFα, and nuclear to Ki‐67. The strong staining of Bcl‐2, VEGFα, and Ki‐67 was present in 92.6%, 91.6%, and 94.6%, respectively, in the cervical cancer sample (Tables 2 and 3, and Figure 2A–C). There was an association between the higher expression of Bcl‐2 with the higher expression of STAT3, NF‐κB, and CD204 (*p* < 0.05, *p* < 0.001, and *p* < 0.001, respectively), but not with CD163 (*p* = 0.13). We observed that the expression of VEGFα has a statistical correlation with the expression of STAT3 and CD163 (*p* < 0.0001 and *p* < 0.05), respectively. On the other hand, the high expression of Ki‐67 was statistically significant with STAT3 (*p* < 0.001), NF‐κB (*p* = 0.05), CD163 (*p* < 0.05), and CD204 (*p* < 0.05) (Tables 2 and 3).

### 3.7 Gene Expression Profile In Tumor Microenvironment

A genetic analysis of cervical cancer tumor tissue was conducted to assess the expression of STAT3, Snail, PD‐1, HPV16, HPV18, FOXP3, EXPORTIN 5 (XPO5), CXCR4, CXCL12, and CD8 (Figure 2C). The oncogenic markers HPV16 and HPV18 exhibited high expression levels of 71.4 and 48.1, respectively. Genes involved in epithelial‐mesenchymal transition, including STAT3 (17.9), Snail (85.2), CXCL12 (20.1), CXCR4 (298.5), and XPO5 (31.2), showed variable expression (Figure 2C). Notably, the maximum expression values for PD‐1 (8775.9), FOXP3 (1804.5), CXCR4 (298.5), and CD8 (159.9) were among the highest recorded (Figure 2C). Data from public databases indicate an average expression of 63.8 Transcripts Per Million (TPM) for STAT3 and 43.5 pTPM for CXCR4 in cervical cancer. FOXP3 shows an average expression of 4.3 TPM, while PDCD1 (PD‐1) registers between 1.6 and 2.5 TPM. Additionally, XPO5, CXCL12, SNAI1 (Snail), and CD8A (CD8) exhibit average expression levels of 13.8, 9.0, 4.3, and 13.8 TPM, respectively. These data reinforce the relevance of these genes in processes associated with cervical carcinogenesis. It is important to note that HPV16 and HPV18 are viruses (human papillomaviruses), and thus their genes are not part of the human genome analyzed in these databases (Figure 2C).

### 3.8 Correlation Between Gene Expression Of Epithelial‐Mesenchymal Transition, Carcinogenesis, and Immunosuppression

The epithelial‐mesenchymal transition (EMT)‐related genes STAT3 and SNAIL were analyzed alongside other genetic markers in this study (Tables S8 and S9, Supporting Information). High STAT3 expression was significantly correlated with elevated expression of HPV16 (*p* < 0.05), CXCL12 (*p* < 0.0001), CXCR4 (*p* < 0.001), XPO5 (*p* < 0.0001), PD‐1 (*p* < 0.0001), FOXP3 (*p* < 0.0001), and CD8 (*p* < 0.001), but not with HPV18 (*p* > 0.05). Similarly, high SNAIL expression was strongly associated with CXCL12, CXCR4, HPV18, HPV16, and PD‐1 (all *p* < 0.0001), as well as XPO5 (*p* = 0.0001) (Tables S8 and S9, Supporting Information).

The oncogenic HPV16 and HPV18 genes were highly correlated (*p* < 0.0001) and showed significant associations with other studied genes (Tables S8 and S9, Supporting Information). High HPV16 expression was linked to CD8 (*p* < 0.01), CXCL12 (*p* < 0.01), CXCR4 (*p* < 0.0001), XPO5 (*p* < 0.0001), PD‐1 (*p* < 0.05), and FOXP3 (*p* < 0.05), while HPV18 expression correlated with CD8, CXCL12, CXCR4, and PD‐1 (all *p* < 0.0001), XPO5 (*p* < 0.05), and FOXP3 (*p* < 0.001) (Tables S8 and S9, Supporting Information).

Immunosuppressive genes also exhibited significant associations (Tables S8 and S9, Supporting Information). High PD‐1 expression correlated with CD8, CXCL12, CXCR4, and FOXP3 (all *p* < 0.0001) and with XPO5 (*p* < 0.05). FOXP3 was significantly associated with CD8, CXCL12, and CXCR4 (*p* < 0.0001) and with XPO5 (*p* < 0.05). CD8 expression showed a strong correlation with CXCL12 and CXCR4 (all *p* < 0.0001) but not with XPO5 (*p* > 0.05). CXCL12 was highly correlated with CXCR4 (*p* < 0.0001) but showed no association with XPO5 (*p* > 0.05), whereas XPO5 correlated significantly with CXCR4 (*p* < 0.001) (Tables S8 and S9, Supporting Information).

### 3.9 Correlation Between STAT3 Protein and Gene Expression

STAT3 signaling pathway contributes to proliferation, infiltration, dissemination, and metastasis, as well as to immunosuppression in TME. In this sense, a significant association was observed between high STAT3 protein expression and elevated STAT3 gene expression, indicating a pathway highly activated in tumors analyzed (*p* < 0.001) (Table S10, Supporting Information).

### 3.10 Serum Cytokines and Their Correlation with Protein and Gene Expression Patterns

A panel of seven immune cytokines was analyzed in the peripheral blood of 55 cervical cancer patients with tumor recurrence or death to assess systemic tumor response. Cytokine levels were quantified using the BD Cytometric Bead Array (CBA) Human Th1/Th2/Th17 Cytokine Kit and compared with tumor protein and genetic expression profiles (34). The percentages of systemic cytokines detected above the threshold were: IL‐2 (1.8%), IL‐4 (9.1%), IL‐6 (52.7%), IL‐10 (3.6%), TNF (0%), IFN‐γ (3.6%), and IL‐17A (34.5%) (Table S11, Supporting Information).

**Figure 3:**
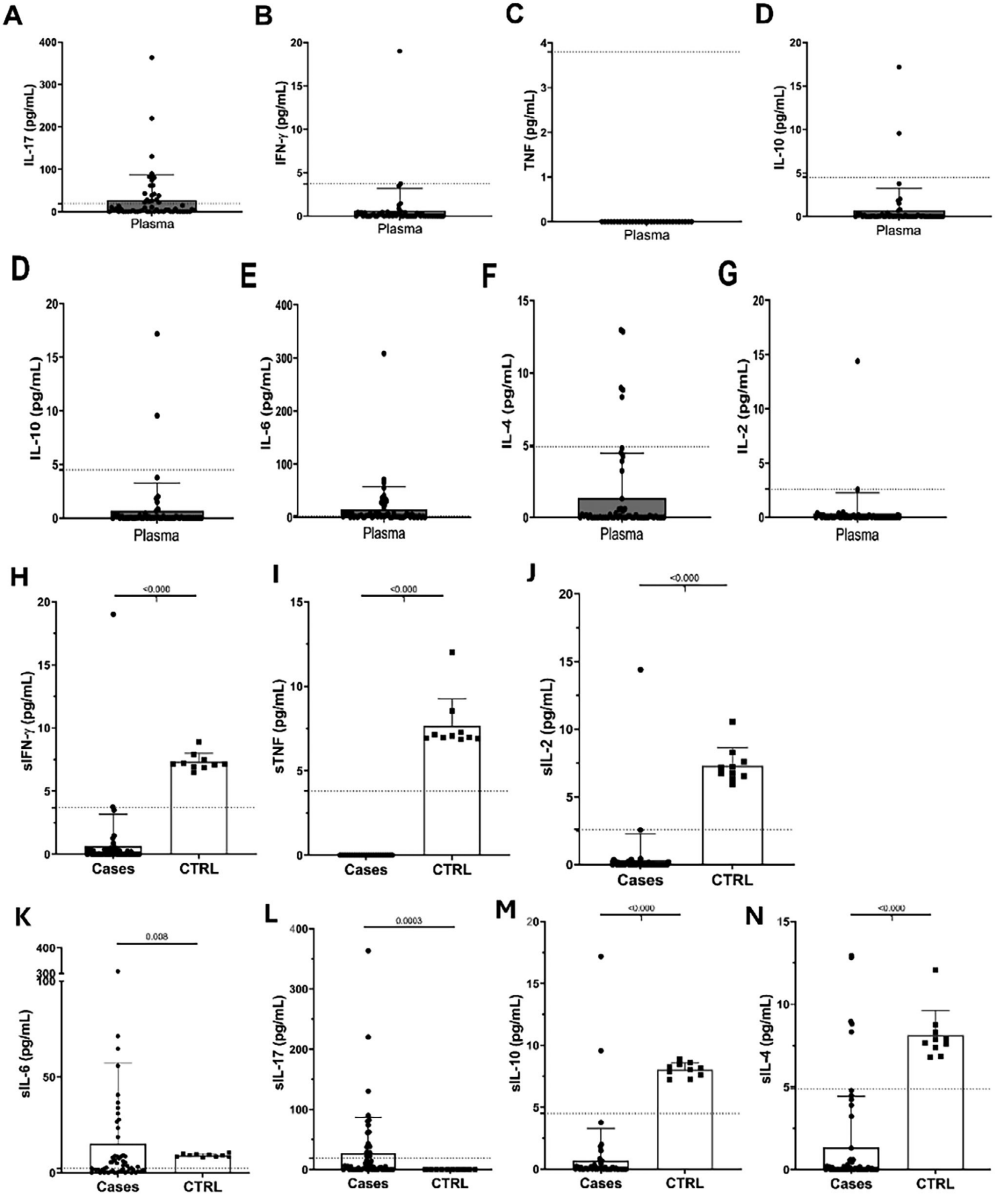
Blood cytokine levels of 55 patients who died or had tumor recurrence. Level of blood cytokines based on the CBA detection limit: A) IL‐17, B) IFN‐γ, C) TNF, D) IL‐10, E) IL‐6, F) IL‐4, and G) IL‐2. Percentage of blood cytokine levels in these patients compared with healthy ones: H) IFN‐γ, I) TNF, J) IL‐2, K) IL‐6, L) IL‐17, M) IL‐10, and N) IL‐4.

Cytokine correlation analysis revealed strong statistical associations for IL‐17A (*p* < 0.001), IL‐6 (*p* < 0.01), and IFN‐γ, TNF, IL‐10, IL‐4, and IL‐2 (all *p* < 0.0001) between patients and healthy controls (**Table** **6** and **Figure** **3**). IL‐2 expression correlated with IL‐6 and IFN‐γ (both *p* < 0.05), while IL‐6 was significantly associated with IL‐17A (*p* < 0.01) (Table 6 and Figure 3).

**Table 6:** Blood cytokine correlations with the gene expression in 55 patients (death or with recurrence).

Marker	Cytokine IL‐17	Cytokine IFN‐γ	Cytokine TNF	Cytokine IL‐10	Cytokine IL‐6	Cytokine IL‐4	Cytokine IL‐2
CD8^*^	r=0.066 *p*=0.69	r=‐0.35 *p*=0.06	r=0.021 *p*=0.92	r=−0.022 *p*=0.46	r=0.060 *p*=0.70	r<0.38 *p* < 0.05	r=−0.33 *p*=0.052
CXCL12^*^	r=0.18 *p*=0.29	r=−0.44 *p*=0.82	r=−0.13 *p*=0.53	r=0.087 *p*=0.63	r=−0.014 *p*=0.93	r=−0.11 *p*=0.52	r=−0.034 *p*=0.85
CXCR4^*^	r=0.29 *p*=0.08	r=−0.004 *p*=0.98	r=0.035 *p*=0.87	r=−0.23 *p*=0.19	r=0.33 *p* < 0.05	r=−0.21 *p*=0.23	r=−0.47 *p*=0.005
XPO5^*^	r=0.11 *p*=0.54	r=0.16 *p*=0.421	r=−0.35 *p*=0.10	r=−0.14 *p*=0.45	r=0.07 *p*=0.68	r=0.21 *p*=0.24	r=0.014 *p*=0.94
FOXP3^*^	r=−0.24 *p*=0.16	r=−0.23 *p*=0.22	r=0.14 *p*=0.52	r=−0.085 *p*=0.64	r=0.06 *p*=0.72	r=−0.30 *p*=0.07	r=−0.49 *p* < 0.01
HPV18^*^	r=0.43 *p*=0.01	r=0.29 *p*=0.12	r=−0.007 *p*=0.93	r=−0.29 *p*=0.11	r=0.14 *p*=0.37	r=−0.004 *p*=0.95	r=−0.27 *p*=0.13
HPV16^*^	r=0.11 *p*=0.56	r=0.48 *p*=0.01	r=−0.33 *p*=0.13	r=−0.16 *p*=0.001	r=−0.56 *p*=0.33	r=0.11 *p*=0.54	r=−0.19 *p*=0.32
PD‐1^*^	r=0.006 *p*=0.97	r=−0.23 *p*=0.20	r=0.20 *p*=0.32	r=−0.14 *p*=0.43	r=0.11 *p*=0.48	r=−0.34 *p* < 0.05	r=−0.36 *p* < 0.05
SNAIL^*^	r=0.001 *p*=0.99	r=0.0006 *p*=0.99	r=0.07 *p*=0.73	r=−0.05 *p*=0.76	r=0.26 *p*=0.87	r=−0.28 *p*=0.09	r=0.22 *p*=0.2
STAT3^*^	r=0.03 *p*=0.87	r=−0.24 *p*=0.24	r=−0.025 *p*=0.91	r=−0.29 *p*=0.15	r=0.14 *p*=0.43	r=−0.15 *p*=0.43	r=−0.34 *p*=0.07
Cytokine IL‐17		r=0.08 *p*=0.64	r=−0.14 *p*=0.47	r=−0.24 *p*=0.19	r=0.47 *p* < 0.01	r=0.23 *p*=0.18	r=−0.10 *p*=0.57
Cytokine IFN‐γ			r=0.13 *p*=0.55	r=−0.09 *p*=0.65	r=−0.22 *p*=0.21	r=0.022 *p*=0.91	r=0.40 *p* < 0.05
Cytokine TNF				r=0.07 *p*=0.75	r=0.08 *p*=0.70	r=−0.02 *p*=0.90	r=0.23 *p*=0.32
Cytokine IL‐10					r=0.25 *p*=0.14	r=−0.16 *p*=0.36	r=0.15 *p*=0.44
Cytokine IL‐6						r=0.07 *p*=0.68	r=−0.35 *p* < 0.05
Cytokine IL‐4							r=−0.10 *p*=0.58

Regarding systemic cytokines and tumor gene expression, high IL‐17A levels were significantly associated with HPV18 (*p* < 0.05). HPV16 expression correlated with IFN‐γ (*p* < 0.05) and IL‐10 (*p* < 0.001), while high IL‐6 levels were linked to CXCR4 (*p* < 0.05). Additionally, IL‐4 was associated with CD8 (*p* < 0.05), and IL‐2 correlated with CXCR4 and FOXP3 (both *p* < 0.01) and PD‐1 (*p* < 0.05) (Table 6 and Figure 3).

Lastly, evaluating the relationship between systemic cytokines and the tumor microenvironment revealed a significant association between IL‐10 and CD163+M2‐TAM expression (*p* < 0.05) (Table S12, Supporting Information).

Significant *p*‐values (*p* < 0.05): Determined using the Spearman correlation test. ^*^Abbreviations: CD8 (lymphocyte receptor), CXCL12 (Chemokine 12), CXCR4 (G‐protein coupled receptor 4), XPO5 (Exportin‐5), FOXP3 (Forkhead box P3), HPV18/16 (Human papillomavirus), PD‐1 (Programmed cell death receptor 1), SNAIL (Snail family), STAT3 (Signal Transducers and Activators of Transcription 3), ILs (Interleukins), TNF (Tumor Necrosis Factor) and IFN‐γ (Interferon‐γ).

### 3.11 Analysis of HLA‐G and FOXP3 Gene Polymorphism in 55 CC Patients who Dead or had Tumor Recurrence

**Figure 4:**
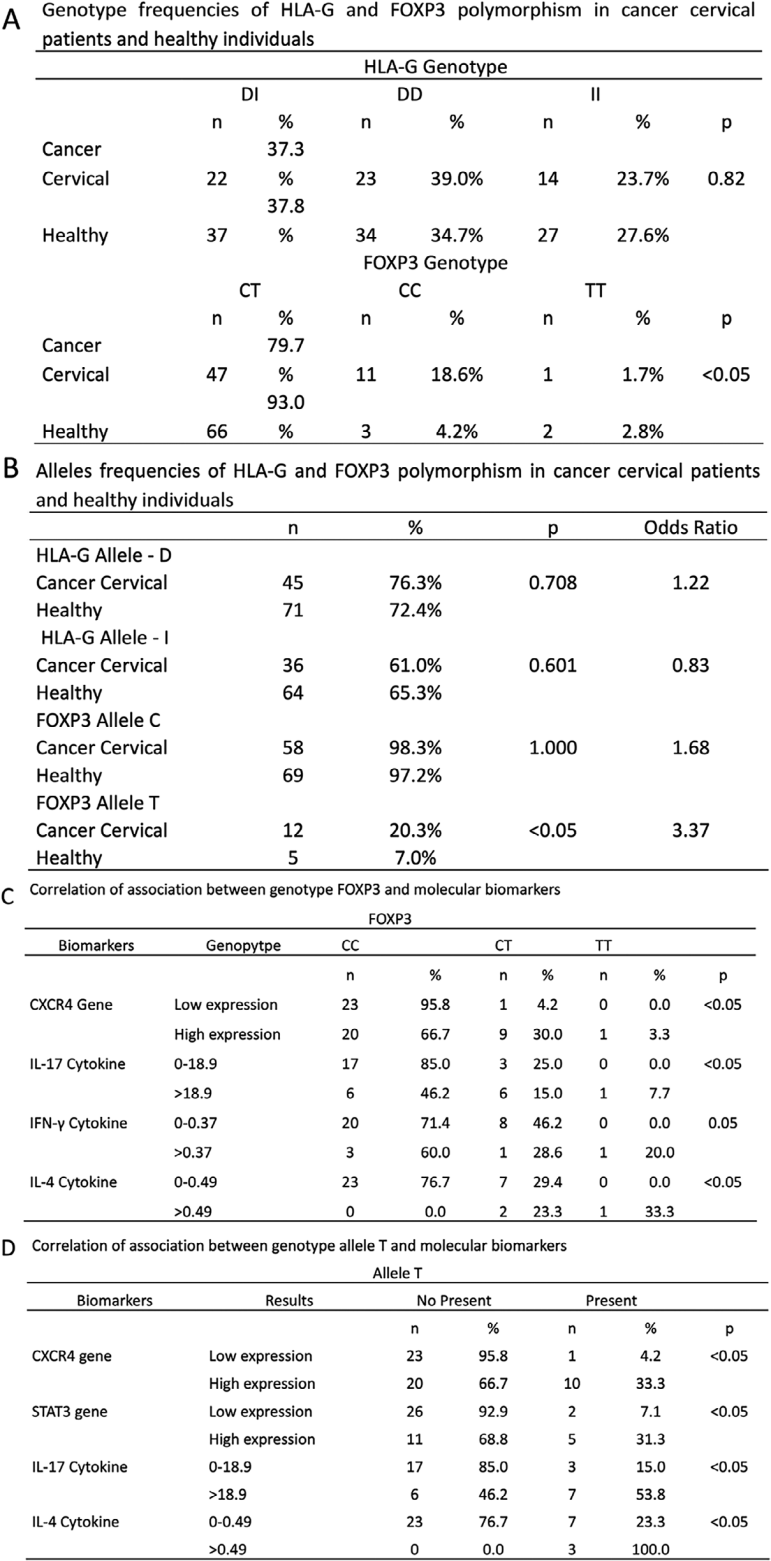
Genetic polymorphism profile of HLA‐G and FOXP3 and its A) Genotype frequencies of HLA‐G and FOXP3 polymorphisms in cervical cancer patients and healthy individuals. B) Allele frequencies of HLA‐G and FOXP3 polymorphisms in cervical cancer patients and healthy individuals. C) Correlation of FOXP3 genotypes with CXCR4 gene and blood cytokines. D) Correlation of the FOXP3 T allele with CXCR4 and STAT3 genes, as well as blood cytokines.

Genetic polymorphism of HLA‐G and FOXP3 was assessed in 55 cervical cancer patients with clinical follow‐up, relapse, or death, and compared to 126 healthy controls. The frequency distribution of the HLA‐G 14 bp Ins/Del genotypes in the 3′‐UTR of exon 8 (rs1704) and FOXP3 SNP (rs3761549) is presented in **Figure** **4**A.

For HLA‐G (rs1704), the control group exhibited D allele (72.4%) and I allele (65.3%), distributed as DD (34.7%), DI (37.8%), and II (27.6%). In cervical cancer patients, the D allele (76.3%) and I allele (61.0%) were distributed as DD (39.0%), DI (37.3%), and II (23.7%) (Figure 4A).

For FOXP3 (rs3761549), the control group had C allele (97.2%) and T allele (7.0%), with genotypes CC (4.2%), CT (93.0%), and TT (2.8%). In cervical cancer patients, C allele (98.3%) and T allele (20.3%) were distributed as CC (18.6%), CT (79.7%), and TT (1.7%). Both polymorphisms conformed to the Hardy–Weinberg equilibrium (Figure 4A).

Statistical significance was observed in the FOXP3 genotype, where the DD genotype was more frequent in patients than the DI genotype (*p* < 0.05). However, no significance was found for HLA‐G. Only the FOXP3 T allele showed a strong association with cervical cancer patients, with an expression probability 3.37 times higher than in healthy individuals (OR: 3.37; *p* < 0.05) (Figure 4B).

### 3.12 Correlation of FOXP3 Gene Polymorphism with the Gene Expression of CXCR4 and STAT3, and the Blood Level of Cytokines (IL‐17, INF‐y and IL‐4)

The FOXP3 polymorphism was correlated with previously studied protein, genetic, and cytokine biomarkers. A significant association was observed between high CXCR4 gene expression and the CT allele pair (*p* < 0.05) (Figure 4C). Additionally, IL‐4 (*p* < 0.05), IL‐17 (*p* < 0.05), and IFN‐γ (*p* = 0.05) showed greater significance for the CC allele (Figure 4C). The FOXP3 T allele correlated with high CXCR4 and STAT3 gene expression and IL‐17 cytokine levels below 18.9(all, *p* < 0.05) (Figure 4D).

### 3.13 Clinical characteristics have a Strong Association Correlation with Blood Gene Polymorphism and Protein Expression of STAT3, NF‐kB, and CD204

**Figure 5:**
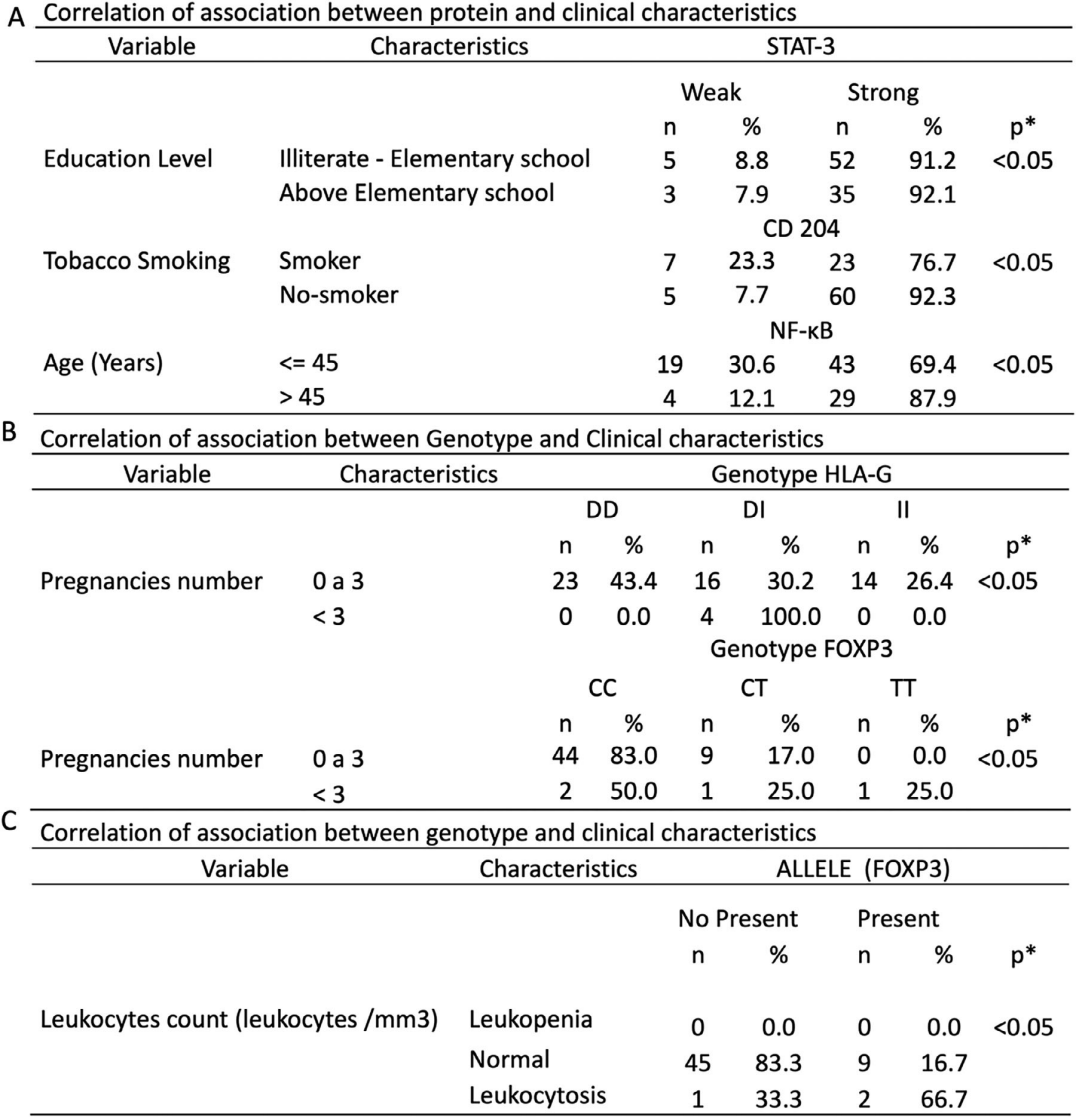
Association Correlation of Blood Gene Polymorphism and Protein expression of STAT3, NF‐kB, and CD204 with clinical characteristics. A) Correlation of STAT3 protein expression with Education level, Smoking, and Age. B) Correlation between HLA‐G Genotype and Pregnancies number. C) Correlation between the Allele T genotype of FOXP3 and Leukocyte count. ^*^ *p* < 0.05, calculated by Chi‐square test.

The clinical characteristics of the studied samples were correlated with biomolecular profiles. A significant association was observed between strong CD204 protein staining and smokers (*p* < 0.05). Patients over 45 years showed a correlation with strong NF‐κB protein staining (*p* < 0.05), indicating increased NF‐κB expression in older individuals. High STAT3 protein expression correlated with higher education levels, suggesting an association between education and STAT3 staining intensity (**Figure** **5**A).

The HLA‐G and FOXP3 polymorphism profile was associated with more than three pregnancies (*p* < 0.05) (Figure 5B). Additionally, the FOXP3 T allele correlated with leukocyte count, where patients with a normal leukocyte count had lower T allele expression than others (*p* < 0.05) (Figure 5C).

## 4 Discussion

In this study, we found that the STAT3/NF‐κB signaling pathways, along with M2‐TAM, play a crucial role in tumor progression, leading to unfavorable outcomes in 100 patients with cervical cancer. There is substantial evidence of complex interactions among stromal cells, such as M2‐TAM, viruses, and tumor cells, mediated by immunosuppressive cytokine loops within the tumor microenvironment (TME).^[^ ^46^ ^]^ Our findings suggest that the upregulation of STAT3/NF‐κB, along with high M2‐TAM infiltration—potentially influenced by lifestyle and clinical factors such as smoking and age (>45 years)—leads to an immunosuppressive TME. This favors cancer hallmarks such as epithelial‐mesenchymal transition (EMT), apoptosis resistance, angiogenesis, and tumor proliferation, impacting systemic immune changes and worsening clinical and pathological prognostic parameters.^[^ ^47^, ^48^, ^49^ ^]^ Intriguingly, we highlight for the first time how survival is affected by the strong correlation between STAT3/NF‐κB pathways, M2‐TAM, oncogenic HPV/XPO5, systemic immunosuppression (via blood immunosuppressive cytokines), and FoxP3/HLA‐G polymorphisms.

Notably, our results suggest a biologically plausible axis where high‐risk HPV infection initiates a cascade of immune dysregulation, leading to activation of STAT3/NF‐κB signaling. These pathways, in turn, upregulate immunosuppressive cytokines (e.g., IL‐6, IL‐10) and drive the polarization of macrophages into the M2 phenotype. This immune profile, influenced by host genetic variants such as FOXP3 and HLA‐G polymorphisms, fosters a tumor microenvironment that favors immune evasion, epithelial‐mesenchymal transition (EMT), and metastasis. The combined action of these mechanisms contributes to the reduced overall survival observed in our cohort.

Certain chemokines have been linked to poor prognosis, not only by promoting metastasis but also by regulating tumor cell proliferation, colonization, angiogenesis, and adhesion to endothelial cells.^[^ ^11^ ^]^ The CXCL12‐CXCR4 chemokine axis is recognized as a key player in cancer progression, particularly through its modulation of SNAIL during EMT.^[^ ^50^, ^51^ ^]^ In our study, higher genetic expression of CXCL12, CXCR4, and SNAIL increased the risk of death by 6.20, 3.90, and 4.64 times, respectively.^[^ ^14^, ^52^, ^53^ ^]^ SNAIL suppresses epithelial markers like E‐cadherin while activating mesenchymal proteins such as N‐cadherin, Vimentin, and matrix metalloproteinases, thereby promoting tumor infiltration, metastasis, and poor prognosis.^[^ ^54^, ^55^, ^56^ ^]^

Our study also demonstrated a strong correlation between the CXCL12‐CXCR4 axis and the STAT3 pathway, SNAIL, FOXP3 polymorphism (T allele), PD‐1, CD8, and systemic cytokines such as IL‐6 and IL‐12, further reinforcing their roles in immunosuppression and metastasis.^[^ ^12^, ^14^, ^57^, ^58^, ^59^, ^60^, ^61^ ^]^ FOXP3, previously described as a suppressor of CD8+ T cells in cervical cancer, facilitates immunosuppressive action in the TME, promoting tumor progression.^[^ ^62^ ^]^ This makes it a potential prognostic and metastatic indicator.^[^ ^63^ ^]^ We propose that FoxP3+ cells likely represent tumor‐specific effector T cells rather than suppressive Tregs.^[^ ^64^ ^]^ The presence of CD4+CD25+ and FoxP3+ Tregs in cervical cancer tumor‐infiltrating lymphocytes (TILs) suggests a negative regulation of effector immunity, opposing immune response activation. Additionally, CD25—a Treg surface receptor—was strongly correlated with IL‐17, VEGFα, FOXP3, and the STAT3/NF‐κB pathways, as well as M2‐TAM markers (CD163/CD204), all associated with poor prognosis.^[^ ^64^, ^65^, ^66^ ^]^

Systemically, HLA‐G overexpression is a known tumor immune evasion strategy, allowing cancer cells to bypass immune surveillance.^[^ ^67^ ^]^ According to Crispim et al. (2015), HLA‐G expression in cervical intraepithelial neoplasia (CIN) was correlated with decreased survival compared to patients with chronic cervicitis.^[^ ^68^ ^]^ HLA‐G expression progressively increased in CIN2/3 and squamous cell carcinoma compared to CIN1, consistent with our findings of higher HLA‐G polymorphism in the blood. This suggests a predisposition to cervical cancer.^[^ ^67^, ^69^ ^]^ Notably, this polymorphism was associated with multiple pregnancies. Specifically, we identified the HLA‐G 3′‐UTR polymorphism (14 bp Ins/Del, RS1704) with a higher DD genotype frequency associated with a higher expression of Treg cells in affected patients, which showed to have a highly immunosuppressive profile.

In the context of immune cell polymorphisms, we also assessed FOXP3 genotype frequencies in patients who relapsed or died. FOXP3 plays a vital role in immune stability and autoimmunity prevention.^[^ ^37^ ^]^ Prior studies have debated the impact of FOXP3 polymorphisms on cancer susceptibility.^[^ ^41^ ^]^ FOXP3 is an immune‐related gene essential for Treg function.^[^ ^22^, ^70^ ^]^ Our findings reveal a significant association between the FOXP3 rs3761549 “T” allele and cervical cancer, suggesting its role as a potential biomarker for disease development and prognosis. More importantly, individuals lacking the FOXP3 T allele (i.e., carrying the CC genotype) showed significantly increased STAT3 pathway expression, elevated systemic IL‐4 and IL‐17 levels, and reduced CD8 gene expression—all features consistent with a pro‐tumoral immunosuppressive profile.

These genetic variants may modulate the host immune response not only by altering cytokine profiles but also by influencing downstream signaling pathways such as STAT3/NF‐κB. For example, the FOXP3 T allele was associated with increased IL‐4 and IL‐17 levels, which are known to promote M2 macrophage polarization via STAT3 activation. Thus, genetic predisposition interacts with immunological signaling to shape the tumor microenvironment in a way that favors progression and immune evasion.

HPV is a well‐established driver of cervical cancer, aiding tumor persistence.^[^ ^71^ ^]^ High‐risk HPV types, particularly HPV16 and 18, contribute to most cervical lesions.^[^ ^9^ ^]^ The HPV genome regulates viral replication and progression, often integrating into host chromosomes and promoting carcinogenesis via viral oncoproteins.^[^ ^8^, ^72^ ^]^ These oncoproteins activate signaling pathways, including STAT3/NF‐κB, thereby inducing immune suppression and facilitating tumor immune evasion.^[^ ^73^, ^74^ ^]^ In our study, HPV16 was detected in 72.9% of patients, coinciding with higher STAT3 and SNAIL expression.

Ki‐67, a nuclear protein associated with cell proliferation, is present in all active cell cycle phases but absent in quiescence (G0).^[^ ^75^ ^]^ We found that elevated Ki‐67 expression strongly correlated with STAT3 pathway upregulation. Notably, microRNAs (miRNAs) are emerging as critical regulators of the cell cycle and cancer biology.^[^ ^76^ ^]^ Altered miRNA expression patterns distinguish tumors from their tissue origins.^[^ ^77^ ^]^ XPO5, a key transporter of precursor miRNAs (pre‐miRNAs) from the nucleus to the cytoplasm, plays a crucial role in miRNA biogenesis.^[^ ^78^ ^]^ Although the clinical significance of XPO5 in cervical cancer remains to be fully elucidated, our findings demonstrate a strong association between its elevated expression and increased activity of STAT3, SNAIL, and high‐risk HPV types (16 and 18), highlighting its potential role in tumorigenesis.

Within the TME, pro‐tumor events are orchestrated by immunosuppressive cytokines that upregulate STAT3/NF‐κB.^[^ ^5^, ^6^, ^7^ ^]^ M2‐TAMs are particularly susceptible to modulation by immunosuppressive environments.^[^ ^5^, ^6^, ^7^ ^]^ Macrophage polarization toward M2‐TAM is driven by cytokines such as IL‐4, IL‐13, and IL‐10, which promote STAT3/NF‐κB activation, enhancing tumor proliferation, infiltration, and metastasis.^[^ ^54^, ^56^, ^79^, ^80^ ^]^ Our study found strong associations between CD163+CD204+ M2‐TAM and STAT3/NF‐κB signaling, as well as tumor proliferation (Ki‐67), dissemination (E‐cadherin), and immunosuppression (MIF).^[^ ^7^ ^]^ Interestingly, weak TGF‐β, IL‐10, and PD‐L1 expression in 66.3%, 69.5%, and 74.7% of patients showed no correlation with M2‐TAM, but lower IL‐10 and PD‐L1 expression were strongly associated with STAT3/NF‐κB pathways, respectively. These weak staining results may limit our ability to fully assess their immunosuppressive roles in mortality. However, alternative pathways likely contribute to tumor progression. Some studies suggest tumor immune escape may involve additional immunosuppressive cytokine loops or immune cell polymorphisms.^[^ ^64^ ^]^

Taken together, our findings reveal a multifactorial interplay in cervical cancer progression, in which viral oncogenesis (via HPV), immune signaling (STAT3/NF‐κB), cellular players (M2‐TAMs, Tregs), and host genetic background (FOXP3 and HLA‐G polymorphisms) operate in concert to drive immune escape and worsen clinical outcomes. This integrated model supports the notion that survival in cervical cancer is not determined by isolated events, but by a dynamic network of molecular and immunogenetic interactions. Personalized immunotherapeutic approaches targeting key nodes of this network—such as STAT3 inhibitors, TAM reprogramming, or checkpoint modulation—may represent promising strategies to improve prognosis in affected patients.

Although our study provided important data on protein and gene expression of cancer hallmarks influencing survival in cervical cancer, a major limitation was the lack of longitudinal assessment during treatment, after therapy, or at recurrence. This was mainly due to logistical and clinical barriers, including recruitment difficulties, technical limitations of repeated tissue sampling, high costs, and anticipated patient refusal rates. Therefore, we restricted our analysis to treatment‐naive patients, ensuring a more uniform baseline. Future studies exploring molecular marker dynamics across different stages of treatment and disease evolution remain highly relevant.

These findings have important implications for cancer treatment, emphasizing the need for personalized immunotherapy based on molecular profiles, including cytokine and immune cell gene polymorphisms. Collectively, our results indicate that M2‐TAM infiltration and upregulated STAT3/NF‐κB pathways drive tumor proliferation, angiogenesis, EMT, and immunosuppression, leading to poor clinical outcomes. Systemic immunosuppressive cytokines and immune cell polymorphisms further exacerbate the condition, resulting in a worse prognosis and shorter survival in cervical cancer patients.

## 5 Conclusion

M2‐TAM, STAT3/NF‐κB, and TME proteins positively regulate tumor remodeling and angiogenesis through immune system interactions, influencing clinical outcomes. Genetic alterations in CXCL12‐CXCR4, SNAIL, FOXP3, and the FOXP3 rs3761549 polymorphism further confirm poor prognosis. We propose that targeting the CXCL12‐CXCR4 axis, SNAIL, FOXP3, and its rs3761549 variant in an oncogenic HPV/XPO5 TME—characterized by high M2‐TAM infiltration and STAT3/NF‐κB upregulation—may help counteract immunosuppression and metastasis in cervical cancer.

FOXP3 polymorphism, along with CXCL12‐CXCR4 axis and SNAIL upregulation in an oncogenic HPV/XPO5 TME with high M2‐TAM infiltration, critically influences cervical cancer patient survival.

## Conflict of Interest

The authors declare no conflict of interest.

## Author Contributions

GAL, FMA, IGS, JCOC, GAL, RSC, COM‐A, NLLM, VES, and RCQA contributed to the experimental techniques and analysis of medical records; GAL, RSC, COM‐A, RNC, and RFAJ analyzed data; GAL, RSC, COM‐A, RNC, and RFAJ and wrote the manuscript; GAL and RFAJ checked the final edition of the manuscript; RFAJ managed the funding and designed this research.

## Supporting information



Supporting Information

## Data Availability

The data that support the findings of this study are available on request from the corresponding author. The data are not publicly available due to privacy or ethical restrictions.

## References

[1] M. Arbyn , E. Weiderpass , L. Bruni , S. de Sanjosé , M. Saraiya , J. Ferlay , F. Bray , Lancet Global Health 2020, 8, 191. 10.1016/S2214-109X(19)30482-6 PMC7025157 31812369

[2] H. Sung , J. Ferlay , R. L. Siegel , M. Laversanne , I. Soerjomataram , A. Jemal , F. Bray , Ca‐Cancer J. Clin. 2021, 71, 209. 33538338 10.3322/caac.21660

[3] M. S. de Oliveira , F. C. L. da Silva , L. F. Leite , J. F. O. Pinto , L. M. de Andrade , M. C. de Camargo , Revista Brasileira de Cancerologia 2023, 69, 2.

[4] S. J. Piersma , Cancer Microenviron. 2011, 4, 361. 21626415 10.1007/s12307-011-0066-7 PMC3234326

[5] R. S. Cavalcante , U. Ishikawa , E. S. Silva , A. A. Silva‐Júnior , A. A. Araújo , L. J. Cruz , A. B. Chan , R. F. de Araújo Júnior , Br. J. Pharmacol. 2021, 178, 2284. 33434950 10.1111/bph.15373 PMC8251773

[6] T. G. de Carvalho , P. Lara , C. Jorquera‐Cordero , C. F. S. Aragão , A. de Santana Oliveira , V. B. Garcia , S. V. de Paiva Souza , T. Schomann , L. A. L. Soares , P. M. da Matta Guedes , R. F. de Araújo Júnior , Biomed. Pharmacother. 2023, 168, 115663. 37832408 10.1016/j.biopha.2023.115663

[7] G. A. Lira , F. M. de Azevedo , I. Lins , I. L. Marques , C. Eich , R. F. de Araujo Junior , Cancers 2024, 16, 2496. 39061137 10.3390/cancers16142496 PMC11275153

[8] A. N. Burchell , R. L. Winer , S. de Sanjosé , E. L. Franco , Vaccine 2006, 24, S52. 10.1016/j.vaccine.2006.05.031 16950018

[9] S. Kanodia , L. M. Fahey , W. M. Kast , Curr. Cancer Drug Targets 2007, 7, 79. 17305480 10.2174/156800907780006869

[10] V. Wootipoom , N. Lekhyananda , T. Phungrassami , P. Boonyaphiphat , P. Thongsuksai , Gynecol. Oncol. 2004, 94, 636. 15350352 10.1016/j.ygyno.2004.03.012

[11] Y. Long , Y. Jiang , J. Zeng , Y. Dang , Y. Chen , J. Lin , H. Wei , H. Xia , J. Long , C. Luo , Z. Chen , Y. Huang , M. Li , J. Cell. Mol. Med. 2020, 24, 3167. 31991051 10.1111/jcmm.14990 PMC7077540

[12] Y. Shimtzu , K. Dobashi , H. Imai , N. Sunaga , A. Ono , T. Sano , T. Hikino , K. Shimizu , S. Tanaka , T. Ishizuka , M. Utsugi , M. Mori , Int. J. Immunopath. Pharmacol. 2009, 22, 43. 10.1177/039463200902200106 19309551

[13] Y. Tian , P. Qi , Q. Niu , X. Hu , Front. Mol. Biosci. 2020, 7, 27. 32185181 10.3389/fmolb.2020.00022 PMC7058927

[14] K. Mortezaee , Life Sci. 2020, 249, 117534. 32156548 10.1016/j.lfs.2020.117534

[15] F. Guo , W. Kong , G. Zhao , Z. Cheng , L. Ai , J. Lv , Y. Feng , X. Ma , Biosci. Rep. 2021, 41, BSR20210217. 10.1042/BSR20203145 PMC8493445 33928349

[16] M. Y. Lee , M. R. Shen , Am. J. Transl. Res. 2012, 4, 1. 22347518 PMC3276374

[17] L. Cao , P. L. Sun , Y. He , M. Yao , H. Gao , Path. Res. Pract. 2020, 216, 152751. 31776057 10.1016/j.prp.2019.152751

[18] S. P. Kerkar , N. P. Restifo , Cancer Res. 2012, 72, 3125. 22721837 10.1158/0008-5472.CAN-11-4094 PMC6327310

[19] H. H. Xu , X. Zhang , H. H. Zheng , Q. Y. Han , A. F. Lin , W. H. Yan , Infectious Agents and Cancer 2018, 13, 42. 30619504 10.1186/s13027-018-0217-2 PMC6311041

[20] A. Lin , W. H. Yan , Mol. Med. 2015, 21, 782. 26322846 10.2119/molmed.2015.00083 PMC4749493

[21] T. Hayashi , K. Yoshikawa , S. Suzuki , M. Gosho , R. Ueda , Y. Kazaoka , Clin. Experim. Dental Res. 2022, 8, 152. 10.1002/cre2.477 PMC8874079 34319010

[22] P. J. Chen , C. W. Lin , H. J. Lu , C. Y. Chuang , S. F. Yang , Y. E. Chou , J. Cancer 2023, 14, 1195. 37215447 10.7150/jca.84470 PMC10197947

[23] S. Li , Y. Shen , C. Dong , S. Yin , D. Zhou , A. Zhou , Cell Investig 2025, 1, 100005.

[24] T. Hideki , S. Muneaki , I. Mitsuya , Y. Nobuo , Japanese J. Clinical Oncol. 2019, 49, 311.

[25] R. F. Araújo Jr. , G. A. Lira , T. Schomann , R. S. Cavalcante , N. F. Vilar , R. C. M. de Paula , R. F. Gomes , C. K. Chung , C. Jorquera‐Cordero , O. Vepris , A. B. Chan , L. J. Cruz , Transl. Oncol. 2023, 30, 101647. 10.1016/j.tranon.2023.101647 PMC9989692 36857852

[26] S. Rohilla , B. Krämer , F. Koberling , I. Gregor , A. C. Hocke , Sci. Rep. 2020, 10, 3820. 32123277 10.1038/s41598-020-60877-8 PMC7052234

[27] B. Moser , B. Hochreiter , R. Herbst , J. A. Schmid , Biotechnol. J. 2017, 12, 1600332. 27420480 10.1002/biot.201600332 PMC5244660

[28] R. F. Araújo Jr. , G. A. Lira , J. A. Vilaça , H. G. Guedes , M. C. A. Leitão , H. F. Lucena , C. C. O. Ramos , Path., Res. Practice 2015, 211, 71. 10.1016/j.prp.2014.09.007 25446246

[29] R. F. Araújo Júnior , V. B. Garcia , R. F. C. Leitão , G. A. C. Brito , E. C. Miguel , P. M. M. Guedes , A. A. Araújo , PLoS One 2016, 11, 0148868. 10.1371/journal.pone.0148868 PMC4758650 26891124

[30] G. Angelico , A. Santoro , F. Inzani , P. Straccia , S. Spadola , D. Arciuolo , M. Valente , N. D'Alessandris , R. Benvenuto , A. Travaglino , A. Raffone , G. F. Zannoni , Diagnostics 2021, 11, 667. 33923427 10.3390/diagnostics11040713 PMC8073999

[31] K. Harada , X. Dong , J. S. Estrella , A. M. Correa , Y. Xu , W. L. Hofstetter , K. Sudo , H. Onodera , K. Suzuki , A. Suzuki , R. L. Johnson , Z. Wang , S. Song , J. A. Ajani , Gastric Cancer 2018, 21, 31. 28801853 10.1007/s10120-017-0760-3

[32] W. Staniszewski , Folia Histochemica et Cytobiologica 2009, 47, 699. 20430741 10.2478/v10042-009-0115-y

[33] S. Fasanella , E. Leonardi , C. Cantaloni , C. Eccher , I. Bazzanella , D. Aldovini , E. Bragantini , L. Morelli , L. Cuorvo , A. Ferro , F. Gasperetti , G. Berlanda , P. Dalla Palma , M. Barbareschi , Diagnostic Pathology 2011, 6, S7. 21489202 10.1186/1746-1596-6-S1-S7 PMC3073225

[34] M. García‐Rojo , J. R. Sánchez , E. de la Santa , E. Durán , J. L. Ruiz , A. Silva , F. J. Rubio , A. M. Rodríguez , B. Meléndez , L. González , B. López‐Viedma , Diagnostic Pathology 2014, 9, S7. 25565117 10.1186/1746-1596-9-S1-S7 PMC4305977

[35] B. R. Barricelli , E. Casiraghi , J. Gliozzo , V. Huber , B. E. Leone , A. Rizzi , B. Vergani , BMC Bioinformatics 2019, 20, 733. 31881821 10.1186/s12859-019-3285-4 PMC6935242

[36] A. Lashen , M. S. Toss , A. R. Green , N. P. Mongan , E. Rakha , Histopathology 2022, 81, 786. 35997652 10.1111/his.14781 PMC9826086

[37] S. Otani , T. Fujii , I. Kukimoto , N. Yamamoto , T. Tsukamoto , R. Ichikawa , E. Nishio , A. Iwata , Cytokine 2019, 120, 210. 31121496 10.1016/j.cyto.2019.05.011

[38] S. Xie , L. Zhu , L. Wang , S. Wang , X. Tong , W. Ni , Oncol. Lett. 2024, 27, 237. 38601181 10.3892/ol.2024.14370 PMC11005083

[39] C. Suwanvecho , L. K. Krčmová , F. Švec , TrAC, Trends Anal. Chem. 2024, 180, 117909.

[40] L. A. Salazar , M. H. Hirata , S. A. Cavalli , M. O. Machado , R. D. Hirata , Clinical Chem. 1998, 44, 1748. 9702967

[41] J. G. N. Gomes , A. P. D. Lima , K. T. C. De Carvalho , J. C. D. O. Crispim , J. Dental Med. Sci. 2016, 15, 39.

[42] M. Ota , H. Fukushima , J. K. Kulski , H. Inoko , Nat. Protoc. 2007, 2, 2857. 18007620 10.1038/nprot.2007.407

[43] Z. Mojtahedi , N. Erfani , M. R. Haghshenas , S. V. Hosseini , A. Ghaderi , Iranian J. Colorectal Res. 2013, 1, 1.

[44] A. Sakakibara , K. Matsui , T. Katayama , T. Higuchi , K. Terakawa , I. Konishi , J. Obstetr. Gynaecol. Res. 2019, 45, 686. 10.1111/jog.13891 30623525

[45] W. Shin , S. Y. Park , S. S. Seo , M. C. Lim , J. Y. Kim , S. Kang , Gynecol. Oncol. 2022, 164, 62. 34696893 10.1016/j.ygyno.2021.10.070

[46] K. Pietras , A. Ostman , Exp. Cell Res. 2010, 316, 1324. 20211171 10.1016/j.yexcr.2010.02.045

[47] C. Murdoch , M. Muthana , S. B. Coffelt , C. E. Lewis , Nat. Rev. Cancer 2008, 8, 618. 18633355 10.1038/nrc2444

[48] J. Condeelis , J. W. Pollard , Cell 2006, 124, 263. 16439202 10.1016/j.cell.2006.01.007

[49] S. Shi , H. Y. Ma , Z. G. Zhang , Pathol., Res. Practice 2021, 227, 153624. 10.1016/j.prp.2021.153624 34571355

[50] Z. Huang , Mini Reviews in Med. Chem. 2002, 2, 373. 10.2174/1389557023405855 12370058

[51] S. Albert , M. E. Riveiro , C. Halimi , M. Hourseau , A. Couvelard , M. Serova , B. Barry , E. Raymond , S. Faivre , Head & Neck 2013, 35, 1819. 23468253 10.1002/hed.23217

[52] W. Zhao , Y. Zhou , H. Xu , Y. Cheng , B. Kong , Clin. Invest. Med. 2013, 36, E223. 23906494 10.25011/cim.v36i4.19956

[53] K. Mortezaee , Life Sci. 2020, 249, 117534. 32156548 10.1016/j.lfs.2020.117534

[54] Q. Wang , A. Steger , S. Mahner , U. Jeschke , H. Heidegger , Int. J. Mol. Sci. 2019, 20, 3310. 31284453 10.3390/ijms20133310 PMC6651300

[55] Á. López‐Janeiro , C. Padilla‐Ansala , C. E. de Andrea , D. Hardisson , I. Melero , Modern Pathology 2020, 33, 1458. 32291396 10.1038/s41379-020-0534-z

[56] Y. Lin , J. Xu , H. Lan , J. Hematol. Oncol. 2019, 12, 76. 31300030 10.1186/s13045-019-0760-3 PMC6626377

[57] X. Sun , G. Cheng , M. Hao , J. Zheng , X. Zhou , J. Zhang , R. S. Taichman , K. J. Pienta , J. Wang , Cancer Metastasis Rev. 2010, 29, 709. 20839032 10.1007/s10555-010-9256-x PMC3175097

[58] J. H. Yen , C. C. Chang , H. J. Hsu , C. H. Yang , H. Mani , J. W. Liou , Tzu Chi Medical Journal 2024, 36, 231. 38993827 10.4103/tcmj.tcmj_52_24 PMC11236080

[59] R. Mezzapelle , M. Leo , F. Caprioglio , L. S. Colley , A. Lamarca , L. Sabatino , V. Colantuoni , M. P. Crippa , M. E. Bianchi , Cancers 2022, 14, 2267. 35565443 10.3390/cancers14092314 PMC9105267

[60] C. D'Alterio , G. Nasti , M. Polimeno , A. Ottaiano , M. Conson , L. Circelli , G. Botti , G. Scognamiglio , S. Santagata , C. De Divitiis , A. Nappi , M. Napolitano , F. Tatangelo , R. Pacelli , F. Izzo , E. Vuttariello , G. Botti , S. Scala , Oncoimmunology 2016, 5, 1254313. 10.1080/2162402X.2016.1254313 PMC5214751 28123896

[61] H. Kulbe , P. Chakravarty , D. A. Leinster , K. A. Charles , J. Kwong , R. G. Thompson , J. I. Coward , T. Schioppa , S. C. Robinson , W. M. Gallagher , L. Galletta , M. A. Salako , J. F. Smyth , T. Hagemann , D. J. Brennan , D. D. Bowtell , F. R. Balkwill , Cancer Res. 2012, 72, 66. 22065722 10.1158/0008-5472.CAN-11-2178 PMC3252703

[62] S. Mishra , S. Srinivasan , C. Ma , N. Zhang , Front. Immunol. 2021, 12, 708874. 34484208 10.3389/fimmu.2021.708874 PMC8416339

[63] C. Loddenkemper , C. Hoffmann , J. Stanke , D. Nagorsen , U. Baron , S. Olek , J. Huehn , J. P. Ritz , H. Stein , A. M. Kaufmann , A. Schneider , G. Cichon , Cancer Sci. 2009, 100, 1112. 19514119 10.1111/j.1349-7006.2009.01153.x PMC11159425

[64] A. M. Heeren , J. Rotman , A. G. M. Stam , N. Pocorni , A. A. Gassama , S. Samuels , M. C. G. Bleeker , C. H. Mom , H. J. M. A. A. Zijlmans , G. G. Kenter , E. S. Jordanova , T. D. de Gruijl , J. Immunotherapy of Cancer 2019, 7, 43. 10.1186/s40425-019-0526-z PMC6373123 30755279

[65] D. Das , B. Sarkar , S. Mukhopadhyay , C. Banerjee , S. B. Mondal , Asian Pacific J. Cancer Prevention, APJCP 2018, 19, 471. 10.22034/APJCP.2018.19.2.471 PMC5980936 29480666

[66] C. O. Sung , W. Park , Y.‐L. Choi , G. Ahn , S. Y. Song , S. J. Huh , D. S. Bae , B. G. Kim , J. H. Lee , Radiotherapy Oncol. 2010, 95, 359. 10.1016/j.radonc.2010.01.007 20153907

[67] H. H. Xu , Y. Y. Xie , G. Jun , Z. Yang , Q. Y. Han , J. Cancer Res. Clin. Oncol. 2023, 149, 4195. 36053326 10.1007/s00432-022-04331-4 PMC10349748

[68] L. N. Miranda , F. P. S. Reginaldo , D. M. B. O. Souza , C. P. Soares , T. G. A. Silva , K. B. F. Rocha , C. A. N. Jatobá , E. A. Donadi , J. M. L. Andrade , A. K. S. Gonçalves , J. C. O. Crispim , São Paulo Medical Journal 2015, 133, 336. 25351636 10.1590/1516-3180.2013.7170009 PMC10876348

[69] P. Li , N. Wang , Y. Zhang , C. Wang , L. Du , Front. Immunol. 2021, 12, 791535. 34868081 10.3389/fimmu.2021.791535 PMC8636042

[70] F. Shi , X. X. Pang , G. J. Li , Z. H. Chen , M. Y. Dong , J. L. Wang , J. Cell. Mol. Med. 2022, 26, 2658. 35322929 10.1111/jcmm.17276 PMC9077298

[71] M. Stanley , Eur. J. Obs. Gynecol. Reproductive Biol. 2006.

[72] C. A. Burmeister , S. F. Khan , G. Schäfer , N. Mbatani , T. Adams , J. Moodley , S. Prince , Tumour Virus Research 2022, 13, 200238. 35460940 10.1016/j.tvr.2022.200238 PMC9062473

[73] N. Fontecha , M. Basaras , S. Hernáez , D. Andía , R. Cisterna , BMC Cancer 2016, 16, 852. 27816058 10.1186/s12885-016-2885-x PMC5097850

[74] M. A. G. Gonçalves , E. A. Donadi , The Brazilian Journal of Infectious Diseases 2004, 8, 1. 15137933

[75] S. F. Yang , S.‐S. F. Yuan , Y. T. Yeh , S.‐C. Hung , M. T. Wu , J. H. Su , C. Y. Chai , Kaohsiung J. Med. Sci. 2006, 22, 539. 17110342 10.1016/S1607-551X(09)70350-X PMC11917641

[76] J. Lu , G. Getz , E. A. Miska , E. Alvarez‐Saavedra , J. Lamb , D. Peck , A. Sweet‐Cordero , B. L. Ebert , R. H. Mak , A. A. Ferrando , J. R. Downing , T. Jacks , H. R. Horvitz , T. R. Golub , Nature 2005, 435, 834. 15944708 10.1038/nature03702

[77] Y. W. Iwasaki , K. Kiga , H. Kayo , Y. Fukuda‐Yuzawa , J. Weise , T. Inada , M. Tomita , Y. Ishihama , T. Fukao , RNA 2013, 19, 490. 23431327 10.1261/rna.036608.112 PMC3677259

[78] J. Ristau , J. Staffa , P. Schrotz‐King , B. Gigic , K. W. Makar , M. Hoffmeister , H. Brenner , A. Ulrich , M. Schneider , C. M. Ulrich , N. Habermann , Cancer Epidemiol., Biomarkers Prev. 2014, 23, 2632. 25472670 10.1158/1055-9965.EPI-14-0556 PMC5699859

[79] R. Huang , S. Wang , N. Wang , Y. Zheng , J. Zhou , B. Yang , X. Wang , J. Zhang , L. Guo , S. Wang , Z. Chen , Z. Wang , S. Xiang , Cell Death Dis. 2020, 11, 234. 32300100 10.1038/s41419-020-2435-y PMC7162982

[80] T. Kusaba , T. Nakayama , K. Yamazumi , Y. Yakata , A. Yoshizaki , K. Inoue , T. Nagayasu , I. Sekine , Oncol. Reports 2006, 15, 1445. 16685378

