# Supplementary material for: FOXP3 Polymorphism and Upregulation of the CXCL12‐CXCR4‐SNAIL Axis with High Infiltration of M2TAM by STAT3/NFKB Pathways Influence the Survival of Cervical Cancer Patients
Source: Adv Biol (Weinh). 2025 Nov 20;9(12):e00354. doi: 10.1002/adbi.202500354 (PMC12712755; doi:10.1002/adbi.202500354)
Supplement: Supplementary file 1 — Supporting Information [file ADBI-9-e00354-s001.docx]

*Supporting Information*

**FOXP3 polymorphism and upregulation of the CXCL12-CXCR4-SNAIL axis with high infiltration of M2TAM by STAT3/NFKB pathways influence the survival of cervical cancer patients**

*George A. Lira*^1^,^2,3,4^, Fábio M. de Azevedo^3,4^, Ingrid G. S. Lins^3^, Janaína C. O. Crispim^5^, Giovanna A. Lira^3^, Rômulo S. Cavalcante*^6^*, Ricardo Cobucci*^7^*, Carolina O. Mendes-Aguiar*^8^*, Rafaela Torres Dantas Da Silva¹^,^², Vinícius E. da Silva¹, Ryan C. Q. Aquino¹, Raimundo F. de Araújo Júnior*^1,2,3^*

^1^ Cancer and Inflammation Research Laboratory, Department of Morphology, Federal University of Rio Grande do Norte Natal, RN 59072-970, Brazil

^2^ Post-Graduation in Health Science, Federal University of Rio Grande do Norte, Natal, RN 59072-970, Brazil

^3^ League Against Cancer from Rio Grande do Norte, Advanced Oncology Center, Natal, RN 59075-740, Brazil

^4^ Pathology Department, Federal University of Rio Grande do Norte, Natal, RN 59012-570, Brazil

^5^ Post-Graduate Program in Technological Development and Innovation in Medicines, Federal University of Rio Grande do Norte, Natal, RN 59072-970, Brazil

^6^ Trairi Faculty of Health Sciences, Federal University of Rio Grande do Norte, Santa Cruz, RN, 59200-000, Brazil.

^7^ Graduate Program of Biotechnology, Potiguar University (UnP), Natal, RN 59056-000, Brazil.

^8^ Laboratory of Immunogenetics of Complex Diseases. Institute of Tropical Medicine of Rio Grande do Norte, UFRN, Natal, RN 59072-970, Brazil

***Correspondence to:**

Prof. Raimundo Fernandes de Araujo Junior, University Campus, Federal University of Rio Grande do Norte, Morphology Department, Brazil. Phone: +558498934310 Email: [fernandes.araujo@ufrn.br](mailto:fernandes.araujo@ufrn.br)

**2. Methods**

**2.1. Tissue samples and data collection**

This research is a prospective study of 100 women diagnosed with cervical cancer in the period from 2018 to 2023 registered in the file of the Advanced Oncology Center of the League Against Cancer of Rio Grande do Norte (LRNCC), Natal, Brazil. Previously, all cases had histopathological confirmation by surgical pathologists of the service, according to the histological types: Squamous Cell Carcinoma, Adenocarcinoma, Adenosquamous Carcinoma and atypical. Initially, 107 patients were selected and 7 were excluded from the study due to the pathological diagnosis of cervical intraepithelial neoplasia or other cancer (n=3), insufficient follow-up (n=1), previous oncological treatment (n=2), lack of data (n=1) or insufficient pathological material (n=1). Likewise, the inclusion criteria adopted to select 100 cases were the following: (I) patients had never undergone any type of oncological treatment, (II) they were scored by the TNM cervical cancer staging system based on the guidelines of the Union for International Cancer Control (UICC) eighth edition^[15]^, (III) patient physically able to collect a tumor and peripheral blood sample, (IV) patients agree to participate in the research by signing the Informed Consent Form (ICF), (V) without surgical contraindication that could affect the prognosis and (VI) with enough surgical material to perform the tissue microarray (TMA). After signing the ICF, biological blood and tumor samples were collected from the women assisted in the outpatient routine of the Gynecology sector of the LRNCC after rigorous clinical, laboratory, cytological and anatomopathological examination (Fig 1). Sociodemographic, clinical health, blood analysis, histopathological and therapeutic data were obtained from individual patient records. In this context, data related to lifestyle, smoking and alcoholism, and patient age at diagnosis were collected. In addition, the histopathological characteristics of the tumors were highlighted, such as degree of cell differentiation, angiolymphatic invasion, neural invasion, depth of stromal invasion, involvement of the tumor margin, lymph node metastases of pelvic regions and retroperitoneum, histological type of cervical cancer, FIGO and TNM stages. Tumor recurrence after treatment, death, number of hemoglobin, leukocytes and platelets were also noted. The Research Ethics Committee (IRB) of the LRNCC, (CAE 2761333, Natal, Brazil) approved this study on May 9, 2018. The patients were treated by the responsible physician of the institution and the clinical conduct performed was in accordance with the LNRCC guidelines.

**2.1.1 Sociodemographic and clinicopathological data**

Sociodemographic, clinical health, blood analysis, histopathological and therapeutic data were obtained from individual patient records. In this context, collected data related to lifestyle, tobacco smoking and alcohol consumption, and patient age at the diagnosis time. Furthermore, histopathology characteristics of tumors were highlighted, such as grade of cellular differentiation, angiolymphatic invasion, neural invasion, depth of stromal invasion, involvement of the tumor margin, lymph node metastasis from pelvic and retroperitoneum regions, histology type of cervical cancer, FIGO and TNM stages. Tumor recurrence after treatment, death, number of hemoglobin, leukocytes, and platelets also were annotated.

**2.3 Double Immunofluorescence Staining**

Immunofluorescence analysis of TMA slides were made as previously described by Araújo Jr. et. al. (2023)^[25]^ and Rohilla S. et. al. (2020)^[26].^ Briefly, after deparaffinization, rehydration and antigenic recovery ( 11 µM sodium citrate solution at 90°C for 30min), the blocking of tissue autofluorescence was performed using 3% hydrogen peroxide and 0.1% Sudan Black. The slides were washed with 0.05 % Tween20 in PBS for 5 minutes three times, the slides were treated in SuperBlock™ (PBS) Blocking Buffer (cat#37580, Thermo Fisher) for 30min. The primary antibodies, STAT3 (1:100) and CD204 (1:100), were diluted in 1% BSA to block nonspecific binding sites. The slides were then incubated at 4°C overnight. The slides were washed with 0.05 % Tween20 in PBS for 5 minutes three times, Then incubated with goat anti-mouse Alexa Fluor 488-conjulgated (1:300) and anti-rabbit Atto 550-conjugated secondary antibodies, nuclear stain was done with Fluoromount-G, with DAPI (cat# 00-4959-52, Thermo Fisher). An Olympus trinocular fluorescence microscope, model BX51TF, series 6F2120 6 with a HD Lite 1080P digital camera was used to analyze the slides. According to fluorescence intensity mean value, cells were considered positive when they exhibited green fluorescence in the membrane and red fluorescence in the cytoplasm. Marker expression was determined using the numerical results obtained from the image analysis software algorithms, which quantified pixel intensity on a scale of 0 to 21.437. Based on this quantification, fluorescence was categorized as follows: scores 1 and 2 corresponded to weak or absent fluorescence (<0.001); score 3 indicated moderate fluorescence (algorithm 0.001–10.718); and score 4 represented intense fluorescence (algorithm 10.719–21.437). ^[24-26]^

**2.4 Quantitative Evaluation of Immunostaining**

Slides were analyzed using Aperio CS2 device (Aperio, Vista, CA, USA) and Image Scope software (Aperio, Vista, CA, USA). Representative areas of each sample were analyzed by electronic morphometry. Necrotic and autolytic areas and loss of areas greater than 10% were discarded to analyze. According to the staining intensity, it was considered positive, with cells-stained brown in the nucleus, and/or cytoplasm, and/or membrane (Table 2S). The expression of markers was considered according to the numerical result of the software image analysis algorithms^[19,20]^ which is based on the RGB color system from a scale of 0 to 255 pixels: for the nuclear positivity pattern, the algorithm used was “Nuclear v9”, for the cytoplasm, the algorithm used was “Positive Pixel Count v9” and for membrane, the algorithm used was “Membrane v9”^[21-23]^. In this line, based on the numerical result of the parameter in pixel, a median between the color intensity and the number of cells with positive staining was obtained and considered as “negative”, “weak” (<5%), “moderate” (5-50%) and “strong” (>50%) when, and negative for unmarked ones. Regarding Ki-67, the quantification of Ki-67 positive cells within the hotspot was calculated by the percentage of positive cells in a fixed area of 0.05mm2 of the sample^[28]^. A hotspot was subjectively defined as the area with the highest density of Ki-67-positive tumor cells identified at 20× magnification compared to areas of surrounding tumor tissue and then at 40× magnification. The mean percentage of Ki-67-positive tumor cells, relative to all tumor cells within the hotspot, was estimated by the percentage of Aperio-positive nuclei. To classify, we categorize Ki-67 into distinct low- and high-proliferating tumors, including up to 15% and greater than 15%, respectively^[28]^.

A total of 100 patients who underwent immunohistochemical marking were divided into two groups: negative or weak for categorization "1", and moderate or strong markings were categorized as "2" for the positive group. Ki-67 was categorized as either 1 for low proliferation or 2 for high proliferation.

**2.5. Cytokine Analysis by flow cytometry**

Results were categorized into two groups: Group 1—values below the minimum detection threshold, and Group 2—values within the standard curve (Table 3S).

**2.8. Follow-up**

The study group consists of biopsy of 100 women with cervical cancer followed up from 2018 to 2023. 39 of them made surgery for the histopathological investigation of HPV, angiolymphatic invasion, neural invasion, depth of stromal invasion, involvement of the tumor margin and lymph node metastasis from pelvic and retroperitoneum regions. 62 cases had chemotherapy, 73 cases had external radiotherapy, 67 cases had internal radiotherapy, and 61 cases had radio and chemotherapy. Overall survival (OS) was calculated as the length of time from the date of diagnosis or the start of treatment for a disease (a total of 100 patients), such as cancer, that patients diagnosed with the disease are still alive ^[32,33]^ – a total 67 alive and 33 deaths.

1. **Results**
   1. **Epidemiologic and Clinicopathologic features of the 100 cervical cancer cases**

In total, 100 (100%) patients exhibited an average 47.17 years (Table 5S). We also found the most common level of education for illiterate and elementary school in 62 (62.0%) patients, the predominant race was brown (87%). Regarding the number of managements, 90.9% had up to 3 pregnancies (mean of 4.40 and standard deviation of 3.55). Regarding smoking and alcoholism, 35% are smokers and 36.0% are alcoholics. The vast majority (81.0% and 87.0%) are in a situation of normal hemoglobin and leukocytes, respectively (table 6S). Firstly, the histological type was analyzed and, 69 (69.0%) were classified as squamous cell carcinoma, 22 (22.0%) as adenocarcinoma and 9 (0.8%) as adenosquamous carcinoma or atypical. When the patients were studied about the histological grade of differentiation and aggressiveness of their tumors, 47.0% of them were poorly differentiated, 34 (34.0%) patients were stage I, 21 (21.0%) were stage II, 44 (44.0%) were stage III or IV, and 1 (1.0%) had no staging information. It was also verified that 30.0% of the women died and 22% had recurrence. 39 patients were treated initially by surgery, of which 7 (17.94%) had angiolymphatic invasion, 9 (23.07%) had neural invasion and 21 (79.48%) had full depth of stromal invasion. Lymph node metastasis was found in 12 (30.76%) patients, the average of resected retroperitoneal lymph nodes was 2.5, the mean number of pelvic lymph nodes removed in the right and left iliac fossa was 3.4 and 3.2, respectively, with 1 lymph node being positive in both sides. In other side, found a mean number of pelvic lymph node removed in the right and left obturator fossa of 4.3 and 3.4, respectively, with 6 and 2 positives lymph nodes. Epidemiologic and Clinicopathological characteristics of 100 patients are summarized in Table 6S.

Of the patients treated with adjuvant radiation, 73 patients (73.0%) were treated with external radiation and 67 (67.0%) with internal radiation. While 62 (62.0%) patients underwent chemotherapy.

**3.2 Overall survival to age, epidemiologic data, laboratory analysis and clinicopathological features**

However, survival did not show a statistically significant correlation with age, certain epidemiological factors, or some laboratory and clinicopathological parameters (Tables 3 and 5S).

**3.9 Correlation between STAT3, NF-κB, CD204 and CD163 proteins and gene expression**

The main axis of the M2-TAM with STAT3 and NF-κB has direct action and intercommunication between the signaling pathways that influence the mesenchymal epithelium transition, immunosuppression, tumor proliferation, angiogenesis, and apoptosis in favor of tumor progression, infiltration, dissemination and metastasis. Therefore, we analyzed the CD204+ or CD163+M2-TAM, STAT3 and NF-κB proteins with gene expressions. Thus, we found an association between the strong marking of the STAT3 protein and the high expression of the STAT3 gene with p<0.001, but without any other association with the studied genes such as HPV16, HPV18, CXCL12, CXCR4, XPO5, PD-1, FOXP3 and CD8. As for NF-κB, CD163 and CD204 proteins there was no association with gene expressions (Table 8S).

**3.10. Serum cytokine profiling: Correlations between IL-2, IL-4, IL-6, IL-10, TNF, IFN-γ, and IL-17A with protein and gene expression patterns**

Lastly, evaluating the relationship between systemic cytokines and the tumor microenvironment revealed a significant association between IL-10 and CD163+M2-TAM expression (p<0.05) (Table 10S).

**3.14 Correlation of association between the profile of gene and protein expressions, profile of systemic protein cytokines and clinical staging TNM**

According to exact chi-square test, this article analyzed the biomolecular expressions studied with clinical staging TNM that was found correlation of association to NF-κB protein in tumor and blood IL-17, p<0.05. However, the correlation was not significant for other biomarkers. (Table 11S).

**3.15 Correlation of association between clinicopathological characteristics and pathological TNM stage**

The pathological staging corresponds to the best evaluation of the evolution of the cancer locally, with this the correlation between the pathological TNM and the pathological characteristics was carried out, which showed us a correspondence for lymphatic invasion and the Depth of stromal invasion with p significant for <0.05. Stage III - IV has a significantly higher percentage of total stromal invasion compared to the rest (Table 12S).

**3.16 Prognostic significance of patients by TNM clinic stating**

Clinical staging by TNM is important to categorize the presentation of cancer at the time of diagnosis, which will serve as a therapeutic and prognostic approach. Observing the grouping of patients by TNM staging and the relationship with death through overall survival was found significantly statistical correlation. The overall survival curve showed a significantly statistical with p <0.001 (χ2= 22.13) (Table 12S, 13S and 15S, and fig. 1S).

**3.17 Overall survival evaluation of patients with cervical cancer based on life-style database, laboratory analysis, pathologic stage, clinicopathological characteristics, and treatment.**

In this investigation, we evaluated the analysis of lifestyle database, laboratory analysis, pathologic stage, clinicopathological characteristics, and treatment with death that had statistically significant to hemoglobin count (p<0.05; χ2= 7.36), pathologic stage (p<0.001; χ2= 24.73), degree of pathological differentiation (p<0.05; χ2= 7.48) and surgery (p<0.05; χ2= 7.48) (Table 14S and 16S, and fig. 2S).

Supplementary Tables and Figures

| **Table 1S.** Specification, clone, dilution, antigenic recovery, and incubation time of the primary antibodies. | | | | | |
| --- | --- | --- | --- | --- | --- |
| Antibody | Manufacturer | Clone | Dilution | Antigen retrieval | Incubation |
| Anti-Vimentin | Flex dako – Denmark | Monoclonal mouse –v9 | Flex | Trilogy, 1:100, pascal, 30 min | 60 minutes |
| Anti-NF-kB | Santa Cruz Biotech  P65(a) sc-109 -lot 1007 | Polyclonal - IgG | 1:400 | Trilogy, 1:100, pascal, 30 min | Overnight |
| Anti-TGF ß1 | Santa Cruz Biotech  (V): sc-146-lot G0114 | Polyclonal – IgG  Rabbit | 1:200 | Trilogy, 1:100, pascal, 30 min | Overnight |
| Anti-PD-L1 | Bioisb inc Cd274 | Rbt – pd-l1  Bsb 2652 | 1:100 | Trilogy, 1:100, pascal, 30 min | 60 minutes |
| Anti-E-cadherin | Flex dako  Nch- 38 | Monoclonal Antihuman | Flex | Trilogy, 1:100, pascal, 30 min | 60 minutes |
| Anti-Mif | Santa Cruz Biotech  Fl-115 sc-20121 | Polyclonal Rabbit | 1:200 | Trilogy, 1:100, pascal, 30 min | 60 minutes |
| Anti-VEGF α | Boster Biological Tech  Pa1080 | 100 ug - Lot n010  Human-mouse-rat | 1:500 | Trilogy, 1:100, pascal, 30 minutes | Overnight |
| Anti-CD25 | Invitrogen  Ma51268 | Anticorpo  Il2r1 | 1:200 | Trilogy, 1:100, pascal, 30 minutes | Overnight |
| Anti-CD163 | Proteintech  16646-1-AP | Rabbit  PolyAb | 1:200 | Trilogy, 1:100, pascal, 30 minutes | 60 minutes |
| Anti-IL 10 | Santa Cruz Biotech  Sc-1783 | M18  cabra  Polyclonal | 1:200 | Trilogy, 1:100, pascal, 30 minutes | Overnight |
| Anti-Ki-67 | Dbs  Sp6 | Monoclonal antiki-67 rabbit | 1:200 | Trilogy, 1:100, pascal, 30 minutes | 60 minutes |
| Anti-Stat 3 | Santa Cruz biotech  F-2 -Sc1783 | Monoclonal mouse igg1k | 1:200 | Trilogy, 1:100, pascal, 30 minutes | Overnight |
| Anti-Snail | Santa Cruz Biotech  Sc271977 | G6 | 1:250 | Trilogy, 1:100, pascal, 30 minutes | Overnight |
| Anti-CD204 | Invitrogen  J5htr3 | Antihhuman  Cd204 | 1:200 | Trilogy, 1:100, pascal, 30 minutes | Overnight |
| Anti-FOXP3 | Santa Cruz Biotech  (2A11G9): Sc-53876 | Antihuman | 1:100 | Trilogy, 1:100, pascal, 30 minutes | Overnight |
| Anti-MMP 9 | Boster  Pb10008 | Polyclonal | 1:100 | Trilogy, 1:100, pascal, 30 minutes | Overnight |
| Anti-BcL-2 | Dako | 124 | 1:200 | Trilogy, 1:100, pascal, 30 minutes | 60 minutes |
| Anti-IL 17 | Santa Cruz Biotech  Sc7927 (h132) | Polyclonal | 1:200 | Trilogy, 1:100, pascal, 30 minutes | Overnight |
| Anti-P16 | Roche -  P16 (E6H4) histo - CINtec® p16 | mouse monoclonal anti-p16 | Flex | Automatic System | 24 minutes |

| **Table 2S**. Positivity pattern of antibody cell expression. | | | | | | |
| --- | --- | --- | --- | --- | --- | --- |
| Antibody | Cellular Expression Pattern | Iavg maximum | Iavg minimum | Interval  (weak) | Interval (moderate) | Interval  (strong) |
| Anti-Vimentin | Cytoplasmic / Membrane | 184.3 | 54.4 | 184.3 – 177.8 | 177.8 – 119.4 | 119.4 – 54.4 |
| Anti-NF-kB | Cytoplasmic | 186.1 | 61.78 | 186.1 – 179.9 | 179.9 – 123.9 | 123.9 – 61.78 |
| Anti-Tgf ß1 | Cytoplasmic | 173.95 | 99.94 | 173.95 – 170.24 | 170.24 – 136.94 | 136.94 – 99.94 |
| Anti-PD-L1 | Cytoplasmic | 183.61 | 113.02 | 183.61 – 180.08 | 180.08 – 148.42 | 148.32 – 113.02 |
| Anti-E-cadherin | Cytoplasmic / Membrane | 206.3 | 55.71 | 206.3 – 198.77 | 198.77 – 131.00 | 131.00 – 55.71 |
| Anti-Mif | Cytoplasmic | 163.98 | 51.03 | 163.98 – 158.33 | 158.33 – 107.50 | 107.50 – 51.03 |
| Anti-VEGF α | Cytoplasmic | 155.34 | 56.85 | 155.34 – 150.42 | 150.42 – 106.10 | 106.10 – 56.85 |
| Anti-CD25 | Cytoplasmic | 180 | 84,3 | 180 – 175 | 175 – 132 | 132 – 84.3 |
| Anti-CD163 | Cytoplasmic | 211 | 89.52 | 221 – 202.92 | 204.92 – 150.26 | 150.26 – 89.52 |
| Anti-IL 10 | Cytoplasmic | 186 | 103 | 186 – 181 | 181 – 144 | 144 - 103 |
| Anti-Stat 3 | Cytoplasmic / Nuclear | 198 | 36 | 198 – 189.99 | 189.99 – 117 | 117 - 36 |
| Anti-Snail | Nuclear | 207.00 | 107.00 | 207.00 – 202.00 | 202.00 – 157.00 | 157.00 – 107.00 |
| Anti-CD204 | Cytoplasmic / Nuclear | 152.2 | 86.2 | 152.2 – 148.92 | 148.92 – 119.21 | 119.21 – 86.2 |
| Anti-FoxP3 | Cytoplasmic | 204.8 | 87.57 | 204.8 – 198.9 | 198.9 – 146.2 | 146.2 – 87.57 |
| Anti-MMP9 | Cytoplasmic / Nuclear | 170.25 | 49.34 | 170.25 – 164.2 | 164.2 – 109.8 | 109.8 – 49.34 |
| Anti-Bcl-2 | Cytoplasmic / Nuclear | 182 | 72.34 | 182.00 – 176.51 | 176.51 – 127.17 | 127.17 – 72.34 |
| Anti-IL 17 | Cytoplasmic | 163.5 | 82.27 | 163.5 – 159.43 | 159.43 – 122.88 | 122.88 – 82.27 |
| Anti-Ki-67* | Nuclear | 1 | 96 |  | | |
| Anti-P16 | Cytoplasmic / Nuclear | Positive | Negative |  | | |

| *Analyzed by percent positive nuclei in hotspot (%) |
| --- |
| Positive – Brown color and Negative – Blue color. |

| **Table 3S.** Cytokine Limit of detection (pg/mL). | | |
| --- | --- | --- |
| Cytokine | Group 1 - Below Standard Curve | Group 2 – On the standard Curve |
| IL-2 | < 2.6 | > 2.6 |
| IL-4 | < 4.9 | > 4.9 |
| IL-6 | < 2.4 | > 2.4 |
| IL-10 | < 4.5 | > 4.5 |
| TNF | < 3.8 | > 3.8 |
| IFN-γ | < 3.7 | > 3.7 |
| IL-17A | < 18.9 | > 18.9 |

| **Table 4S.** Target, primer number, primers sequence, and code of RT-PCR. | | | |
| --- | --- | --- | --- |
| Target | Number | Sequence | Code |
| HPV16 E2F | I3337A11 | GCCGTCAACCAGTCCATAGG | [NM-004399.3](https://www.ncbi.nlm.nih.gov/entrez/viewer.fcgi?db=nucleotide&id=1676440381) |
| HPV16 E2R | I3337A12 | GAGCACTACGCTGGCAAAATC | [NM-004399.3](https://www.ncbi.nlm.nih.gov/entrez/viewer.fcgi?db=nucleotide&id=1676440381) |
| HPV18 E2F | I3337B01 | TTTTCTGGGCCAGCCTTTAG | [NM-130839.5](https://www.ncbi.nlm.nih.gov/entrez/viewer.fcgi?db=nucleotide&id=1772785840) |
| HPV18 E2R | I3337B02 | GTTGCACGAAGGTCCCTTTC | [NM-130839.5](https://www.ncbi.nlm.nih.gov/entrez/viewer.fcgi?db=nucleotide&id=1772785840) |
| SnailF | I3337B03 | CCCCAATCGGAAGCCTAACT | [NM-005985.4](https://www.ncbi.nlm.nih.gov/entrez/viewer.fcgi?db=nucleotide&id=1519243938) |
| SnailR | I3337B04 | GCTGGAAGGTAAACTCTGGATTAGA | [NM-005985.4](https://www.ncbi.nlm.nih.gov/entrez/viewer.fcgi?db=nucleotide&id=1519243938) |
| STAT3F | I3337B05 | GCGCCTGCGATGTCTCA | [NM-006099.3](https://www.ncbi.nlm.nih.gov/entrez/viewer.fcgi?db=nucleotide&id=115298685) |
| STAT3R | I3337B06 | GGAAACTCATCACCATGTGCTTT | [NM-006099.3](https://www.ncbi.nlm.nih.gov/entrez/viewer.fcgi?db=nucleotide&id=115298685) |
| FOXP3F | I3337B11 | CACCTGGCTGGGAAAATGG | [NM-014009.4](https://www.ncbi.nlm.nih.gov/entrez/viewer.fcgi?db=nucleotide&id=1732746273) |
| FOXP3R | I3337B12 | GGAGCCCTTGTCGGATGAT | [NM-014009.4](https://www.ncbi.nlm.nih.gov/entrez/viewer.fcgi?db=nucleotide&id=1732746273) |
| CXCL12F | I3337C01 | GCCGCCACTGCCTTCA | [NM-199168.4](https://www.ncbi.nlm.nih.gov/entrez/viewer.fcgi?db=nucleotide&id=1677556841) |
| CXCL12R | I3337C02 | GGAGCCCACAGAGCCAATC | [NM-199168.4](https://www.ncbi.nlm.nih.gov/entrez/viewer.fcgi?db=nucleotide&id=1677556841) |
| CXCR4F | I3337C05 | CATGGAACCGATCAGTGTGAG | NM-009911.3 |
| CXCR4R | I3337C06 | TGAAGGCCAGGATGAGAACG | NM-009911.3 |
| CD8F | I3337C07 | CCTTGGCCGGGACTTGT | [NM-001768.7](https://www.ncbi.nlm.nih.gov/entrez/viewer.fcgi?db=nucleotide&id=1813796442) |
| CD8R | I3337C08 | TGTGGTTGCAGTAAAGGGTGAT | [NM-001768.7](https://www.ncbi.nlm.nih.gov/entrez/viewer.fcgi?db=nucleotide&id=1813796442) |
| PD-1 (CD279)F | I3337D01 | CAACACATCGGAGAGCTTCGT | [NM-005018.3](https://www.ncbi.nlm.nih.gov/entrez/viewer.fcgi?db=nucleotide&id=1519242236) |
| PD-1 (CD279)R | I3337D02 | AAGGCGGCCAGCTTGTC | [NM-005018.3](https://www.ncbi.nlm.nih.gov/entrez/viewer.fcgi?db=nucleotide&id=1519242236) |
| Exportin (XPO5)F | I3337D03 | TTGGGAGCCGCTGAGTGT | [NM-020750.3](https://www.ncbi.nlm.nih.gov/entrez/viewer.fcgi?db=nucleotide&id=1519315705) |
| Exportin (XPO5)R | I3337D04 | GCTTCCGGTCTTCCAACTTG | [NM-020750.3](https://www.ncbi.nlm.nih.gov/entrez/viewer.fcgi?db=nucleotide&id=1519315705) |

| **Table 5S.** Distribution of 100 patients with cervical cancer by age, clinical stage, recurrence, and survival. | | | |
| --- | --- | --- | --- |
| Clinicopathological features | Category | Frequency | Percentage (%) |
| Age (Years) | <= 45 | 63 | 63.0 |
|  | > 45 | 37 | 37.0 |
|  | Total Number | 100 | 100.0 |
| TNM Staging System | I | 34 | 34.0 |
|  | II | 21 | 21.0 |
|  | III + IV | 44 | 44.0 |
|  | Not available | 1 | 1.0 |
|  | Total Number | 100 | 100.0 |
| Recurrence | No | 78 | 78.0 |
|  | Yes | 22 | 22.0 |
|  | Total Number | 100 | 100.0 |
| Death | No | 67 | 67.0 |
|  | Yes | 33 | 33.0 |
|  | Total Number | 100 | 100.0 |

| **Table 6S.** Distribution of patients by lifestyle database, laboratory analysis, clinical stage, treatment, and clinicopathological. | | | |
| --- | --- | --- | --- |
| Variable | Charactheristics | Frequency | Percentage |
| Pregnancies number | 0 - 3 | 90 | 90.0% |
|  | > 3 | 9 | 9.0% |
|  | Not available | 1 | 1.0% |
|  | Total | 100 | 100.0% |
| Sexual Partners number | < 5 | 62 | 62.0% |
|  | > = 5 | 25 | 25.0% |
|  | Not available | 13 | 13.0% |
|  | Total | 100 | 100.0% |
| Tobacco Smoking | Smoker | 35 | 35.0% |
|  | No-smoker | 65 | 65.0% |
|  | Total Number | 100 | 100.0% |
| Alcohol consumption | Alcoholic | 36 | 36.0% |
|  | No-alcoholic | 64 | 64.0% |
|  | Total | 100 | 100.0% |
| Hemoglobin count (g/dL) | Until 10 | 19 | 19.0% |
|  | > 10 | 81 | 81.0% |
|  | Total | 100 | 100.0% |
| Leukocytes count (leukocytes / | Leukocytosis | 10 | 10.0% |
| mm^3^) | Normal | 87 | 87.0% |
|  | Leukopenia | 2 | 2.0% |
|  | Not available | 1 | 1.0% |
|  | Total | 100 | 100.0% |
| Treatment of surgery | No | 61 | 61.0% |
|  | Yes | 39 | 39.0% |
|  | Total Number | 100 | 100.0% |
| Histological type | [Squamous cell carcinoma](https://www.cancer.gov/Common/PopUps/popDefinition.aspx?id=46595&version=patient&language=English&dictionary=Cancer.gov) | 69 | 69.0% |
|  | [Adenocarcinoma](https://www.cancer.gov/Common/PopUps/popDefinition.aspx?id=46216&version=patient&language=English&dictionary=Cancer.gov) | 22 | 22.0% |
|  | [Adenoescamoso](https://www.cancer.gov/Common/PopUps/popDefinition.aspx?id=46216&version=patient&language=English&dictionary=Cancer.gov) and atypical | 9 | 9.0% |
|  | Total | 100 | 100.0% |
| Angiolymphatic Invasion* | No | 31 | 79.48% |
|  | Yes | 7 | 17.94% |
|  | Not available | 1 | 2.56% |
|  | Total | 39 | 100.0% |
| Neural Invasion* | No | 29 | 74.35% |
|  | Yes | 9 | 23.07% |
|  | Not available | 1 | 2.56% |
|  | Total | 39 | 100.0% |
| Degree of pathological | Well differentiated | 16 | 16.0% |
| differentiation | Moderately differentiated | 34 | 34.0% |
|  | Little differentiated | 47 | 47.0% |
|  | Not available | 3 | 3.0% |
|  | Total | 100 | 100.0% |
| Depth of stromal invasion* | 1/3 internal | 12 | 30.76% |
|  | 2/3 internal | 5 | 12.82% |
|  | Total | 21 | 53.84% |
|  | Not available | 1 | 2.56% |
|  | Total | 39 | 100.0% |
| Lymph node metastasis* | Negative | 26 | 66.66% |
|  | Positive | 12 | 30.76% |
|  | Not available | 1 | 2.56% |
|  | Total | 39 | 100.0% |
| TNM Pathological Staging* | I | 25 | 64.10% |
|  | II | 2 | 5.12% |
|  | III - IV | 12 | 30.76% |
|  | Total | 39 | 100.0% |
| External Radiation Therapy | No | 27 | 27.0% |
|  | Yes | 73 | 73.0% |
|  | Total | 100 | 100.0% |
| Internal Radiation Therapy | No | 33 | 33.0% |
|  | Yes | 67 | 67.0% |
|  | Total | 100 | 100.0% |
| Chemotherapy | No | 38 | 38.0% |
|  | Yes | 62 | 62.0% |
|  | Total | 691 | 100.0% |
|  | | | |

*The assessment was based on the 39 patients with cervical cancer who underwent surgery

Pregnancies number – Average: 4,40 (DP 3,55); Min: 1; Max: 19.

| **Table 7S**. Cox-regression for overall survival to lifestyle, laboratory analysis, of clinical stage, clinicopathological characteristic and treatment. | | | | | |
| --- | --- | --- | --- | --- | --- |
| Variable | Characteristics | Freq* | Median  (CI 95%), mo** | *X^2^* | *p**** |
| Age (year) | >= 45 | 63 | 40.5 (37.0-43.0) | 0.095 | 0.758 |
|  | > 45 | 37 | 36.0 (27.4-44.0) |  |  |
| Education Level | Illiterate - Elementary school | 62 | 40.0 (36.0-43.0) | 0.003 | 0.955 |
|  | Above Elementary school | 38 | 40.0 (35.0-45.0) |  |  |
| Race | White | 9 | 33.0 (7.0-53.0) | 1.841 | 0.398 |
|  | Brown | 87 | 40.0 (36.0-43.0) |  |  |
|  | Black | 4 | 37.5 (34.0-41.0) |  |  |
| Sexual Partners number | < 5 | 62 | 40.0 (36.0-43.0) | 0.341 | 0.559 |
|  | > = 5 | 25 | 40.0 (31.0-46.0) |  |  |
| Pregnancies number | 0 - 3 | 90 | 40.0 (36.0-43.0) | 1.488 | 0.222 |
|  | > 3 | 9 | 40.5 (10.0-54.0) |  |  |
| Tobacco Smoking | Smoker | 35 | 42.0 (36.0-46.0) | 0.030 | 0.862 |
|  | No-smoker | 65 | 40.0 (36.0-42.0) |  |  |
| Alcohol consumption | Alcoholic | 36 | 42.0 (36.0-45.0) | 0.021 | 0.884 |
|  | No-alcoholic | 64 | 39.0 (36.0-42.0) |  |  |
| Hemoglobin count (g/dL) | Until 10 | 19 | 41.0 (32.0-47.0) | 7.369 | **<**0.05 |
|  | > 10 | 81 | 40.0 (36.0-42.0) |  |  |
| Leukocytes count | Leukopenia | 10 | 52.0 ( ) | 4.390 | 0.111 |
| (leukocytes /mm3) | Normal | 87 | 40.0 (36.0-43.0) |  |  |
|  | Leukocytosis | 2 | 37.0 (23.0-42.0) |  |  |
| TNM Staging | I | 34 | 40.0 (35.0-46.0) | 22.132 | **<**0.001 |
|  | II | 21 | 40.0 (35.0-430) |  |  |
|  | III - IV | 44 | 40.0 (31.0-43.0) |  |  |
| Treatment of surgery | Yes | 38 | 41.0 (36.0-45.0) | 10.417 | **<**0.05 |
|  | No | 62 | 40.0 (35.0-42.0) |  |  |
| Histological type | [Squamous cell carcinoma](https://www.cancer.gov/Common/PopUps/popDefinition.aspx?id=46595&version=patient&language=English&dictionary=Cancer.gov) | 69 | 40.0 (36.0-43.0) | 5.700 | 0.058 |
|  | [Adenocarcinoma](https://www.cancer.gov/Common/PopUps/popDefinition.aspx?id=46216&version=patient&language=English&dictionary=Cancer.gov) | 22 | 42.5 (36.0-46.0) |  |  |
|  | [Adenoescamoso](https://www.cancer.gov/Common/PopUps/popDefinition.aspx?id=46216&version=patient&language=English&dictionary=Cancer.gov) and atypical | 9 | 30.5 (21.0-39.0) |  |  |
| Lymphatic Invasion**** | Yes | 18 | 38.5 (34.0-46.0) | 0.008 | 0.927 |
|  | No | 20 | 36.5 (27.0-45.0) |  |  |
| Angiovascular Invasion**** | Yes | 7 | 39.0 (35.0-45.0) | 2.508 | 0.113 |
|  | No | 31 | 47.0 (46.0-54.0) |  |  |
| Neural Invasion**** | Yes | 9 | 45.0 (10.0-54.0) | 3.037 | 0.081 |
|  | No | 29 | 37.0 (34.0-46.0) |  |  |
| Degree of pathological | Well differentiated | 16 | 41.5 (24.0-46.0) | 7.489 | **<**0.05 |
| differentiation | Moderately differentiated | 34 | 37.0 (35.0-43.0) |  |  |
|  | Little differentiated | 47 | 39.5 (35.0-44.0) |  |  |
| Depth of stromal invasion**** | 1/3 internal | 12 | 36.0 (21.0-46.0) | 0.711 | 0.701 |
|  | 2/3 internal | 5 | 36.5 (7.0-47.0) |  |  |
|  | Total | 21 | 42.0 (36.0-46.0) |  |  |
| Lymph node metastasis**** | Negative | 26 |  |  |  |
|  | Positive | 12 |  |  |  |
| TNM Pathological | I | 25 | 38.0 (30.0-46.0) | 24.730 | **<**0.001 |
| Staging**** | II | 2 |  |  |  |
|  | III - IV | 12 | 42.0 (10.0-46.0) |  |  |
| External Radiation Therapy | Yes | 27 |  |  |  |
|  | No | 73 |  |  |  |
| Internal Radiation Therapy | Yes | 67 | 40.5 (36.0-43.0) | 1.022 | 0.312 |
|  | No | 33 | 38.5 (35.0-46.0) |  |  |
| Chemotherapy | Yes | 62 | 40.5 (36.0-42.0) | 0.018 | 0.894 |
|  | No | 38 | 38.0 (35.0-46.0) |  |  |
|  | | | | | |

*Freq: Frequency.

**CI, mo: indicates confidence interval, months; *p**= Significant p<0.05 *(X^2^*: Chi-square Test).

***Log Rank (Mantel-Cox).

****The assessment was based on the 38 patients with cervical cancer who underwent surgery.

**Table 8S.** Correlation of association between gene expression of CD8, CXCL12, CXCR4, XPO5 and in cervical cancer.

| GENES | CXCL12 | CXCR4 | XPO5 | FOXP3 | HPV18 | HPV16 | PD-1 | SNAIL | STAT3 |
| --- | --- | --- | --- | --- | --- | --- | --- | --- | --- |
| CD8 | r=0.70  ****p | r=0.57  ****p | r=0.20  p=0.08 | r=0.61  ****p | r=0.50  ****p | r=0.28  *p | r=0.79  ****p | r=0.64  ****p | r=0.35  ***p |
| CXCL12 |  | r=0.92  ****p | r=0.12  p=0.30 | r=0.95  ****p | r=0.95  ****p | r=0.25  *p | r=0.95  ****p | r=0.96  ****p | r=0.63  ****p |
| CXCR4 |  |  | r=0.41  ***p | r=0.61  ****p | r=0.67  ****p | r=0.50  ****p | r=0.63  ****p | r=0.62  ****p | r=0.41  ***p |
| XPO5 |  |  |  | r=0.29  *p | r=0.26  *p | r=0.64  ****p | r=0.2  *p | r=0.41  ****p | r=0.53  ****p |

Spearman correlation test, *p<0.05; **p<0.01; ***p<0.001; ****p<0.0001

**Table 9S.** Correlation of association between gene expression of FOXP3, HPV18, HPV16, PD-1, SNAIL and STAT-3 in cervical cancer (Spearman correlation test, p<0.05).

| GENES | CD8 | HPV18 | HPV16 | PD-1 | SNAIL | STAT-3 |
| --- | --- | --- | --- | --- | --- | --- |
| FOXP3 |  | r=0.41  ***p | r=0.26  *p | r=0.81  ****p | r=0.63  ****p | r=0.53  ****p |
| HPV18 |  |  | r=0.53  ****p | r=0.49  ****p | r=0.57  ****p | r=0.20  *p |
| HPV16 |  |  |  | r=0.27  *p | r=0.44  ****p | r=0.28  *p |
| PD-1 |  |  |  |  | r=0.77  ****p | r=0.50  ****p |
| SNAIL |  |  |  |  |  | r=0.43  ***p |

Spearman correlation test, *p<0.05; **p<0.01; ***p<0.001; ****p<0.0001

| **Table 10S.** Correlation of association between STAT3 protein and STAT3 gene. | | | | | | |
| --- | --- | --- | --- | --- | --- | --- |
|  |  | STAT3 protein | | | |  |
|  |  | Weak | | Strong | |  |
|  |  | n | % | n | % | p |
| STAT3 gene | Low expression | 3 | 6.5 | 43 | 93.5 | <0.001 |
|  | High expression | 2 | 6.9 | 27 | 93.1 |  |
| p = Significant p<0.05 (Chi-square Test). | | | | | | |

| **Table 11S.** Cytokine profile of blood samples of 55 patients (death or with recurrence) based on CBA. | | | |
| --- | --- | --- | --- |
| Cytokine | Limit of detection (pg/mL) | n | % |
| IL-17 (n=55) |  |  |  |
| No detection | 0-18.9 | 36 | 65.5 |
| Detection | > 18.9 * | 19 | 34.5 |
| IFN (n=55) |  |  |  |
| No detection | 0-3.7 | 53 | 96.4 |
| Detection | > 3.7 * | 2 | 3.6 |
| TNF (n=55) |  |  |  |
| No detection | 0-3.8 * | 55 | 100.0 |
| Detection | > 3.8 * | 0 | 0.0 |
| IL-10 (n=55) |  |  |  |
| No detection | 0-4.5 | 53 | 96.4 |
| Detection | > 4.5 * | 2 | 3.6 |
| IL6 (n=55) |  |  |  |
| No detection | 0- 2.4 | 26 | 47.3 |
| Detection | > 2.4 * | 29 | 52.7 |
| IL4 (n=55) |  |  |  |
| No detection | 0-4.9 | 50 | 90.9 |
| Detection | > 4.9 * | 5 | 9.1 |
| IL2 (n=55) |  |  |  |
| No detection | 0-2.6 | 54 | 98.2 |
| Detection | > 2.6 * | 1 | 1.8 |
|  | | | |

*Above limit of detection.

| **Table 12S.** Correlation of association between CD163 protein and IL-10 blood cytokine. | | | | | | |
| --- | --- | --- | --- | --- | --- | --- |
|  |  | CD163 | | | |  |
|  |  | Weak | | Strong | |  |
|  |  | n | % | n | % | *p* |
| IL-10 Cytokine | 0-4.5 | 16 | 30.8 | 36 | 69.2 | <0,05 |
|  | > 4.5 | 2 | 100.0 | 0 | 0.0 |  |
|  | | | | | | |

*p* = Significant p<0.05 (Chi-square Test).


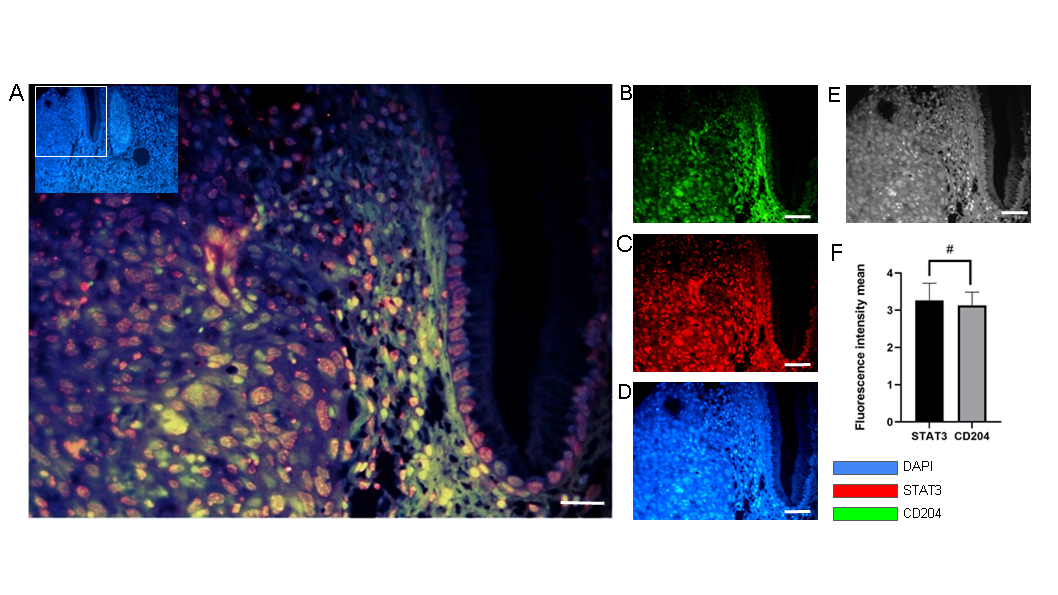
 **Figure 1 S**. Representative images of moderate/strong double immunofluorescence staining with anti-STAT3 and anti-CD204 antibodies on TMA sections from 56 cervical cancer patients. **A)** Representation of all 3 channel of a fluorescently stained core; B**)** Green the M2-TAM marker (CD204). C**)** Red representing the STAT3 marker (STAT3), and **D)** blue the DAPI channel highlighting the cell nuclei; **E)** isolation of tissue autofluorescence; F**)** Quantitative co-expression of STAT3 and CD204 in the TME of 56 patients with CC. scale bars 5 μm. # no statistical significance.
